# Differential Expression of IgM and IgD Discriminates Two Subpopulations of Human Circulating IgM^+^IgD^+^CD27^+^ B Cells That Differ Phenotypically, Functionally, and Genetically

**DOI:** 10.3389/fimmu.2020.00736

**Published:** 2020-05-06

**Authors:** Diana Bautista, Camilo Vásquez, Paola Ayala-Ramírez, Juan Téllez-Sosa, Ernestina Godoy-Lozano, Jesús Martínez-Barnetche, Manuel Franco, Juana Angel

**Affiliations:** ^1^Instituto de Genética Humana, Facultad de Medicina, Pontificia Universidad Javeriana, Bogotá, Colombia; ^2^Centro de Investigación Sobre Enfermedades Infecciosas, Instituto Nacional de Salud Pública, Cuernavaca, Mexico

**Keywords:** memory B cells, marginal zone B cells, cell surface molecules, cell proliferation, gene expression, Ig gene repertoire, human, blood

## Abstract

The origin and function of blood IgM^+^IgD^+^CD27^+^ B cells is controversial, and they are considered a heterogeneous population. Previous staining of circulating B cells of healthy donors with rotavirus fluorescent virus-like particles allowed us to differentiate two subsets of IgM^+^IgD^+^CD27^+^: IgM^hi^ and IgM^lo^ B cells. Here, we confirmed this finding and compared the phenotype, transcriptome, *in vitro* function, and Ig gene repertoire of these two subsets. Eleven markers phenotypically discriminated both subsets (CD1c, CD69, IL21R, CD27, MTG, CD45RB, CD5, CD184, CD23, BAFFR, and CD38) with the IgM^hi^ phenotypically resembling previously reported marginal zone B cells and the IgM^lo^ resembling both naïve and memory B cells. Transcriptomic analysis showed that both subpopulations clustered close to germinal center-experienced IgM only B cells with a Principal Component Analysis, but differed in expression of 78 genes. Moreover, IgM^hi^ B cells expressed genes characteristic of previously reported marginal zone B cells. After stimulation with CpG and cytokines, significantly (*p* < 0.05) higher frequencies (62.5%) of IgM^hi^ B cells proliferated, compared with IgM^lo^ B cells (35.37%), and differentiated to antibody secreting cells (14.22% for IgM^hi^ and 7.19% for IgM^lo^). IgM^hi^ B cells had significantly (*p* < 0.0007) higher frequencies of mutations in IGHV and IGKV regions, IgM^lo^ B cells had higher usage of *IGHJ6* genes (*p* < 0.0001), and both subsets differed in their HCDR3 properties. IgM^hi^ B cells shared most of their shared IGH clonotypes with IgM only memory B cells, and IgM^lo^ B cells with IgM^hi^ B cells. These results support the notion that differential expression of IgM and IgD discriminates two subpopulations of human circulating IgM^+^IgD^+^CD27^+^ B cells, with the IgM^hi^ B cells having similarities with previously described marginal zone B cells that passed through germinal centers, and the IgM^lo^ B cells being the least differentiated amongst the IgM^+^CD27^+^ subsets.

## Introduction

Many subsets of B cells are currently recognized that play numerous central roles in human health (1, 2). The memory B cells (Bmem cells) subset that expresses IgM seem particularly interesting, because they protect against specific pathogens, like encapsulated bacteria (3), but may also be an important source of long-lived memory and thus a key target of vaccines (2). We became interested in the IgM Bmem cells subset when our studies and those of others showed that most rotavirus (RV) specific Bmem cells (RV-Bmem cells) circulating in healthy adults express IgM (4, 5). RV is a ubiquitous intestinal pathogen of humans and animals, and since worldwide almost all children by the age of 2 years have been infected with this virus (6) all adults have circulating RV-Bmem cells. When we adoptively transferred total IgM^+^CD27^+^ B cells purified of healthy adult donors to NOD/Shi-scid interleukin-2 receptor-deficient [IL-2Rγ(null)] immunodeficient mice that were subsequently infected with RV, the B cells performed IgG class-switching and reduced RV viremia and antigenemia (7), indicating that IgM^+^CD27^+^ B cells play a key role in controlling systemic viral dissemination. We further showed that RV-B cells circulating in healthy donors are enriched in IgM^hi^IgD^lo^CD27^+^ rather than in IgM^lo^IgD^hi^CD27^+^ B cells (7, 8). Whether these two subsets differ phenotypically, functionally, and genetically is unknown.

Human circulating IgM^+^IgD^+^CD27^+^ B cells are considered a heterogeneous population (9–15), probably composed at least by B cells of the marginal zone (MZB cells) of the spleen circulating in peripheral blood (13, 16) and by IgM Bmem cells with an adaptive function that entered the germinal centers (17, 18). However, it is debatable if these cells are generated from germinal center responses or independently of T-cell help (14, 16, 18, 19). Most MZB cells studies have been performed in mice and significant anatomical differences between MZ of mice and humans are established (20, 21). However, the characterization of human MZB cells and several features that differentiate them from conventional human follicular naïve B cells and Bmem cells have been recently delineated (13, 14, 21–24). MZB cells, generally characterized as IgM^+^IgD^+^CD27^+^, are the major B cells population in highly specialized structures called marginal zones (MZ) that classically surround the follicles in the spleen, tonsils, and gut-associated lymphoid tissue (GALT) (21, 24). Some studies have proposed that MZB cells are derived from CD27^–^IgM^hi^CD45RB^hi^(MEM55)MTG^±^ transitional (T3’) B cells named MZ precursors cells (MZPc) – present in spleen, blood, and GALT – by engagement of NOTCH2 signaling pathway (24–27). However, others suggest these MZPc are derived from CD27^–^IgM^hi^ transitional (T2) B cells (13, 28). The privileged anatomical position of MZB cells in spleen probably allows them to quickly respond to blood pathogens with an innate-like function (13, 21). To perform their innate-like function, MZB cells respond through a germinal center and T-cell-independent pathway involving the B cells antigen receptor in conjunction with the transmembrane activator and CAML interactor (TACI) receptor and Toll-like receptors (TLR), the latter two preferentially expressed on these B cells (23, 29).

The MZB cells in other organs – GALT, tonsils, or activated lymph nodes – are less well characterized, and their function is unclear (25). It has been recently proposed that MZB cells in intestine diversify their repertoire of immunoglobulins (Ig) in germinal centers of GALT to later travel, via the blood, to the spleen to fulfill their function (24, 30). Indeed, IgM^+^IgD^+^CD27^+^ B cells present in the MZ of the intestine and appendix show clonal relationships with germinal center B cells of these organs and with those that recirculate in blood with the same phenotype (24).

A key issue for the study of human MZB cells has been the identification of the role of developmental and age timing on B cells diversity (31, 32). In children, both spleen and blood IgM^+^IgD^+^CD27^+^ B cells show a pre-diversified Ig gene repertoire generated independently of antigen exposure and of germinal centers (13, 16): children with CD40 ligand deficiency (that lack germinal centers) (19) have conserved numbers of putative MZB cells IgM^+^IgD^+^CD27^+^ in blood, with the expected rate of mutations. Additionally, healthy children present CD27^–^CD45RB^+^ MZPc in spleen and blood that give rise to MZB cells through the NOTCH2 signaling pathway, and patients with a deficiency in this pathway, with Alagille syndrome, show a reduction of these B cells (26, 27). Thus, in children, the evidence supports the notion that MZB cells develop separately from naïve B cells, have innate-like characteristics, and circulating IgM^+^IgD^+^CD27^+^ B cells are related to MZB cells.

In healthy adults, it has also been proposed that MZB cells circulate in the blood as IgM^+^IgD^+^CD27^+^ B cells (13, 26) and are, like in children, phenotypically related to CD27^–^CD45RB^+^ MZPc that circulate in adult blood (24, 26). However, contrary to the evidence in children, the innate-like nature of circulating IgD^+^IgM^+^CD27^+^ B cells of healthy adults is controversial (12): these cells respond to T cell stimuli similarly to class-switched Bmem cells (33) and have mutations in Bcl6, a germinal center marker (18), suggesting that at least a fraction of them are T-cell and germinal center-dependent. Besides, circulating IgM^+^IgD^+^CD27^+^ B cells of healthy adults have a gene expression profile similar to that of IgM only and class-switched Bmem cells (33). Despite this, another study showed differences in the expression of several genes (*SOX7*, *HOXP*, *TOX*, and *COCH*) between IgM^+^IgD^+^CD27^+^ B cells and class-switched Bmem cells (26). Analysis of the Ig gene repertoire of these B cells also delivered inconsistent results: on the one hand, one study found that circulating IgM^+^IgD^+^CD27^+^ Bmem cells use similar *IGHV* gene segments as IgM only and class-switched Bmem cells, and strong clonal relationships of this population with IgM only Bmem cells were identified (17). These findings suggest that the IgM only and class-switched Bmem cells are developmentally related to the IgM^+^IgD^+^CD27^+^ Bmem cells. On the other hand, other studies established that circulating IgM^+^IgD^+^CD27^+^ B cells have differences in the use of *IGHV* gene segments with IgM only Bmem cells (22) and class-switched Bmem cells (34), and strong clonal relationships with class-switched Bmem cells were unexposed in both studies. These results suggest that a large proportion of IgM^+^IgD^+^CD27^+^ B cells derive from a different developmental pathway as class-switched Bmem cells. Given that there is no consensus on the origin of these B cells, we refer to IgM^+^IgD^+^CD27^+^ as B cells instead of Bmem cells. The discrepancies reported in these works are probably explained by the existence of a heterogeneous population of circulating IgM^+^IgD^+^CD27^+^ B cells (9, 10) generated by gradual recruitment over time of MZB cells into germinal center responses (35).

Based on our work with RV-Bmem cells (7), we hypothesized that circulating IgM^+^IgD^+^CD27^+^ B cells are composed of at least two subpopulations (IgM^hi^IgD^lo^ and IgM^lo^IgD^hi^). In the present report, we compared the phenotype, transcriptome, *ex vivo* function, and Ig gene repertoire of circulating IgM^hi^IgD^lo^ and IgM^lo^IgD^hi^ B cells from healthy adults. These results support the notion that differential expression of IgM and IgD discriminates two subpopulations of human circulating IgM^+^IgD^+^CD27^+^ B cells, with the IgM^hi^ B cells having similarities with MZB cells that entered germinal centers and the IgM^lo^ B cells being the least differentiated amongst the IgM^+^CD27^+^ subsets.

## Materials and Methods

### Samples and Cell Sorting

B cells were obtained from buffy coats of healthy adult donors older than 18 years from the Hemocentro Distrital, Bogotá, Colombia, that signed informed consent form approved by the ethics committee of the Pontificia Universidad Javeriana. PBMCs were separated by Ficoll-Hypaque and B cells were enriched by negative selection using the RosetteSep enrichment kit (Stem cell Technologies, Vancouver, BC, Canada#15024). Cells were then stained with LIVE/DEAD^TM^ Fixable Aqua Dead Cell Stain Kit (Invitrogen Molecular Probes, Waltham, MA, United States # L34957) and subsequently with the following previously tittered anti-human monoclonal antibodies (all from BD Bioscience, San Jose, CA, United States, unless otherwise specified): CD3 (V500, clone UCHT1), CD14 (V500, clone M5E2), CD19 (APC-H7, clone SJ25C1), CD27 (PE-CF594, clone M-T271), CD38 (AF-700, clone HIT2), IgD (VB421, clone IA6-2), and IgM (PerCPCy5.5 clone G20-127). Five subpopulations were obtained by means of cell sorting by fluorescence (FACS): naïve B cells (IgM^+^IgD^+^CD27^–^), IgM only (IgM^+^IgD^–^CD27^+^), and class-switched Bmem cells (IgM^–^IgD^–^CD27^+^) as biological controls, along with IgM^hi^ (IgM^+ +^IgD^+^CD27^+^) and IgM^lo^ (IgM^+^IgD^+ +^CD27^+^) B cells (Supplementary Figures 1A,B). Between 150.000 and 1.000.000 total cells were obtained per subpopulation. All cell subpopulations obtained had a post sorting purity greater than 95% (Supplementary Figure 3). Plasmablasts (CD19^+^CD38^+ +^CD27^+ +^) were excluded from the analysis. Samples were sorted on a FACS ARIA IIu (BD Bioscience, San Jose, CA, United States) (36).

### Phenotype Analysis of B Cells Subpopulations

PBMCs were separated by Ficoll-Hypaque from healthy adult donors that signed an informed consent form and stained with antibodies described in section “Samples and Cell Sorting.” Additionally, samples were stained with anti-human monoclonal antibodies (BD Bioscience, San Jose, CA, United States): CD40 (PeCy7, clone 5C3), TLR9 (PE, clone eB72-1665), CD95 (PE, clone DX2), CD73 (PE, clone AD2), CD21 (PE, clone Bly-4), IL-21-R (PE, clone 17A12), CD45RO (APC, clone uch1), CD11c (APC, clone S-HCL-3), BAFF-R (APC, clone 11C1), CD43 (APC, clone eBio84-3C1), PD-1 (PE, clone J105), IL2-Rβ-CD122 (PE, clone Mik-β3), CD24 (PE, clone ML5), CD1c (PE, clone L161), CD184 (APC, clone 1265), CD69 (PE-Cy7, clone FN50), CD23 (APC, clone EBVC5-5), CD11b (AF-488, clone ICRF44), CD5 (PE, clone UCHT2), and CD45RB (PE, clone MEM55) for 30 min on ice. For ABCB1 transporter expression experiments PBMC were incubated with MTG at 100 nM (Thermo Fisher Scientific, Waltham, MA, United States #M7514) for 30 min at 37°C (37). Phenotype analysis was done on five unsorted B cells subpopulations described before. For all markers analyzed, a fluorescence minus one (FMO) was included as a negative control during standardization of the staining protocol (36). Samples were acquired on a LSR Fortessa or FACs ARIA II (BD Bioscience) and analyzed using FlowJo v.9.3.2. The data were represented as an integrated mean fluorescent intensity (iMFI = percentage of positive cells × MFI), a metric measure that combines the percentage of cells expressing a marker) and the MFI of the same marker. Non-parametric tests were used for comparisons.

For some experiments, frequencies of RV Bmem cells were determined as previously reported (7). Green fluorescent protein (GFP) coupled to RV VP6/VP2 virus-like particles (VLP) were generated as previously described (38, 39). Total B cells were washed once with PBS, 2% fetal bovine serum, 0.02% sodium azide, and then incubated with 0.8 μg/test GFP-VLPs for 45 min at 4°C in the dark (7).

### Stimulation of Sort Purified B Cells

Sort purified B cells subsets were labeled with carboxyfluorescein succinimidyl ester (CFSE) (40). B cells (0.4–1.0 × 10^6^ cells/ml) were washed twice with sterile PBS and stained with CFDA-SE (Cell-TraceTM CFSE Cell Proliferation Kit, Invitrogen Molecular Probes, Waltham, MA, United States # C34554) for 5 min at room temperature, protected from light. After being washed three times with 10 ml of PBS FBS 5%, naïve B cells subpopulations were stimulated with 2.5 μg/ml of CpG (ODN 2006; InvivoGen, San Diego, CA, United States), 10 ng/ml human recombinant IL–2, 10 ng/ml human recombinant IL–6, 15 ng/ml IL–10 (all cytokines from PreproTech, Rocky Hill, New Jersey), and NIH 3T3 murine fibroblasts (ATCC, Manassas, VA, United States). The NIH 3T3 feeder cells were irradiated with 3,000 rads (Radiotherapy unit, Centro Javeriano de Oncología, Bogotá) and then used at a concentration of 5.000 cells/well. B cells (20.000 in 200 μl of complete medium with 10% FBS) were cultured at 37°C with 5% CO_2_ in flat bottom 48 well plates for different periods. At the end of the cultures, cells were washed twice with sterile PBS and stained on ice with LIVE/DEAD^TM^ Fixable Aqua Dead Cell Stain Kit (Invitrogen Molecular Probes, Waltham, MA, United States # L34957) and subsequently with antibodies (all from BD Bioscience, San Jose, CA, United States) against (labeled): CD19 (APC-H7), CD38 (PerCp-Cy5.5), and CD27 (PE-CF594). After washing, cells were resuspended in 100 μl PBS, 0.5% BSA (Sigma-Aldrich) plus 250 μl of cytofix/cytoperm (BD Pharmingen) and incubated for 20 min at 4°C. After washing twice with perm/wash (BD Pharmingen) cells were stained with antibodies against IgM APC (polyclonal serum, Jackson ImmunoResearch) or IgG APC (clone G18-145, BD Bioscience, San Jose, CA, United States) for 30 min on ice. Samples were acquired on an LSR Fortessa (BD Bioscience, San Jose, CA, United States) and analyzed using FlowJo v.9.3.2 (FlowJo, LLC, OR, United States). Differences among groups were determined using Kruskal-Wallis test followed by a Wilcoxon signed-rank test using GraphPad Prism software (36).

### RNA Isolation and qRT-PCR

Sort purified B cells from each subpopulation (naïve B cells: 800.000–1.000.000, IgM^lo^ B cells: 325.000–380.000, IgM^hi^ B cells: 410.000–500.000, IgM only Bmem cells: 172.000–220.000, and class-switched Bmem cells: 700.000–1.000.000) were recovered in cold TRIZOL (Invitrogen Molecular Probes, Waltham, MA, United States # 15596026). RNA was extracted using the RNeasy micro kit (Qiagen GmbH, Hilden, Germany # 74004). Samples were re-suspended in RNase-free water and their purity and integrity were verified by spectrophotometry in nanodrop 2000/2000c (Thermo Fisher Scientific, Waltham, MA, United States # ND-2000) and electrophoresis using Bionalyzer 2100 (Agilent Technologies, Santa Clara, CA, United States # G2939BA) and RNA pico 6000 kit (Agilent Technologies, Santa Clara, CA, United States # 5067-1513). Only RNA samples with a RNA integrity number (RIN) greater than 7 were used.

The relative expression of *HOPX* and *SOX7* was determined from sorted B cells. qRT-PCR for the human transcripts was performed with specific TaqMan gene expression assays *HOPX* (Hs041888695_m1), *SOX7* (Hs00846731_s1), and *B2M* (Hs00984230_m1) as a housekeeping gene, all by designed by Applied Biosystems (CA, United States) (26). Taqman gene expression assays were used at a concentration of 0.6X in a final volume of 10 μl with the Lightcycler 480 RNA Master Hydrolysis probes kit (Roche Basel, Switzerland # 04991885001). The reaction was conducted with an annealing temperature of 60°C in 45 cycles using a LightCycler^®^ Nano instrument (Roche Basel, Switzerland) real time-PCR system. The PCR products were quantified and purified with a Wizard^®^ SV Gel and PCR Clean-Up System (Promega, Madison, WI, United States) and used to generate a calibration curve. Four serial log 10 dilutions ranging from 1 × 10^9^ to 1 × 10^3^ copies/l were employed, obtaining an efficiency of 100%. Each sample was analyzed in duplicate, and a negative control with water, instead of cDNA, was included in each analysis. Standard curve based method for relative expression was utilized (41). Differences among groups were determined with a *p*-value < 0.05 using Kruskal-Wallis test followed by a Wilcoxon signed-rank test in GraphPad Prism software.

### Transcriptomic Gene Expression Profiling

A total of 50 ng of RNA from each B cell subpopulation from the same individuals (3 donors in total) was used for expression analysis of transcripts with the human Clariom D array (Affymetrix, Santa Clara, CA. United States # 902923). Affymetrix Clariom D CEL files were normalized to produce probeset-level expression values using Expression Console (version 1.4.1.46) with the Robust Multiarray Average (RMA) algorithm (42) and the default probesets defined by Affymetrix. Clariom D CEL files were re-normalized to produce Entrez-Gene-specific expression values using the implementation of the Robust Multiarray Average (RMA) [1] in the affy package (version 1.36.1) (43) included in the Bioconductor software suite (version 2.12) (43) and an Entrez Gene-specific probeset mapping (version 17.0.0) from the Molecular and Behavioral Neuroscience Institute (Brainarray) at the University of Michigan (44)^*cpsbib*^^1^. An unsupervised Principal Component Analysis (PCA) was performed using the prcomp R function with expression values that had been z-normalized across all samples (i.e., set to a mean of zero and a standard deviation of one). Before z-normalization, the expression values were adjusted by donor by taking the residuals of linear models created using the lmFit function in the limma package (version 3.14.4), treating donor as a fixed effect. Linear mixed-effects modeling and the associated analysis of variance were performed using the anova.lme function in the nlme package (version 3.1–131). Pairwise differential expression between groups was assessed using Student’s paired two-sample *t*-test, i.e., performing Student’s *t*-test on the coefficients of a linear model created using the lmFit function in the limma package, treating donor as a fixed effect. Correction for multiple hypothesis testing was accomplished using the Benjamini-Hochberg false discovery rate (FDR) (45). All analyses performed using the Affymetrix-normalized data were carried out using the R environment for statistical computing (version 2.15.1).

GSEA (version 2.2.1) (46) was used to identify biological terms, pathways, and processes that are coordinately up- or down-regulated within each pairwise comparison. The Entrez Gene identifiers of the genes interrogated by the array (as obtained from the Entrez-Gene-specific re-normalization) were ranked according to the paired Student t statistic computed between IgM^hi^ and IgM^lo^ B cells. This ranked list was then used to perform pre-ranked GSEA analyses (default parameters with random seed 1234) using the Entrez Gene versions of the Hallmark, Biocarta, KEGG, Reactome, Gene Ontology (GO), transcription factor and microRNA motif, and immunologic signature gene sets obtained from the Molecular Signatures Database (MSigDB), version 6.0 (47).

All analyses performed using the Brainarray-normalized data were carried out using the R environment for statistical computing (version 3.2.3).

### Sequencing of Light and Heavy Chain Immunoglobulin Genes

Total RNA obtained from circulating purified B cells subpopulations (24–156 ng) of three adult donors was amplified using the 5’-RACE-PCR (48, 49). Briefly, an oligodT is added to the initial RNA at the 3’ end and then reverse transcription is performed using the SuperScript III reverse transcriptase (200 U/μl) (Invitrogen Molecular Probes, Waltham, MA, United States # 18080044) in the presence of the primer illuRACE (5’-TCGTCGGCAGCGTCAGATGTGTATAAGAGACAGmGmGrG-3’, 12 pmol/μl). This is a hybrid primer where m means 2’O methyl and rG is a guanine with sugar ribose. Subsequently, the sample is subjected to amplification with a universal primer that binds 5’ of the FpAmpl cDNA (5’-TCGTCGGCAGCGTCAGATGTGTATAAGAGACAGGGG-3’, 5 or 2.5 pmol/μl) and another hybrid containing the sequence of the constant region of IgM (5’-GTCTCGTGGGC TCGGAGATGTGTATAAGAGACAGAAAGGGTTGGGGCGG ATGCACT-3’, 5 pmol/μl) or IgG (5’-GTCTCGTGGGCTCGG AGATGTGTATAAGAGACAGACCGATGGGCCCTTGGTG-3’, 5 pmol/μl). Products obtained from this amplification were purified from gel using the Qiaquick 250 kit (Qiagen GmbH, Hilden, Germany # 28706) and subjected to a second amplification using specific sequence primers for IgM (5’-AAAGGGTTGGGGCGGATGCACT-3’, 5 pmol/μl) or IgG (5’-ACCGATGGGCCCTTGGTG-3’, 5 pmol/μl) in a nested PCR procedure. The products of the second amplification were purified using the AMPure XP PCR purification kit (Beckman coulter, Brea, CA # A63881). For Kappa light chain, a first amplification was used with sequence-specific primers (5’-ACAGATGGTGCAGCCAC-3’, 2.5 pmol/μl) and a second amplification with a first hybrid (5’-GTCTCGTGGGCTCGGAGATGTGTATAAGAGACAGACA GATGGTGCAGCCAC-3’, 2.5 pmol/μl). The enzyme Accuzyme DNA polymerase high fidelity (Bioline, London, United Kingdom # BIO-21051) was used.

The amplification cycles used were the following: first PCR: 95°C 3 min, 20 cycles of 95°C 30 s, 60°C 30 s, 68°C 30 s, and 68°C 5 min. Second PCR: 95°C 3 min, 20 cycles of 95°C 30 s, 56°C 30 s, 68°C 30 s, and 68°C 5 min. In all cases water was used as a negative control.

The PCR products were quantified by fluorometry using the Qubit dsDNA HS Assay kit (Invitrogen Molecular Probes U.S MA Waltham # Q32851) before joining adapter Nextera XT index 24 indices -96 samples (Illumina, San Diego, CA, United States # 15032353). Kappa light (IGK) and heavy chain (IGH) products were joined to different adapters for sequencing two chains at the same time per subset. Adapters were joined using the following PCR protocol: 72°C 3 min, 95°C 30 s, 8 cycles of 95°C 10 s, 55°C 30 s, 72°C 30 s, and 72°C 5 min.

The final sample, at 6 nM, was treated with the MiSeq Reagent Kit v3 (Illumina, San Diego, CA, United States # 15043894) and finally ran on the sequencer MiSeq (Illumina, San Diego, CA, United States # SY-410-1003) in a paired manner.

### Light and Heavy Chain Repertoire Analysis of Immunoglobulin Genes

Raw sequencing reads were subjected to a quality control process using the FastQC program^2^. The Ig gene repertoire of each subpopulation was subsequently reconstructed with IgRec using single reads as input (50). Using the VJ FINDER tool, IgRec filters out contaminating reads by alignment with the Ig germline IMGT database. Also, VJ FINDER filters out reads that do not fully cover V(D)J region since their error correction can result in false inferences. Large clusters (sequences with more than 5 reads) were used as proxy of lineages and were analyzed with the IMGT/HighVQuest tool^3^ (51, 52), to obtain the following metrics: (1) # of productive IGH and IGK chain sequences; (2) % of mutations in the IGHV and IGKV regions (corresponding to FR1, CDR1, FR2, CDR2, FR3, and part of the CDR3, according to IMGT/HighVQuest); (3) % of *IGHV* and *IGKV* gene and family usage; (4) % *IGHJ* and *IGKJ* gene usage. The HCDR3 length, the relative frequency (number of reads per lineage/total reads in lineages ≥ 5 reads), N1 and N2 length, P (P3’V, P5’D, P3’D, and P5’J) length, and % identity of IGHV-IGHJ with the corresponding germline gene were used as input to perform a Principal Component Analysis (PCA) to compare the different subpopulations using the Orange3 v3.24.1 software (53). Additionally, the length and other properties of HCDR3 and KCDR3 were determined using the *Peptides* package in R^4^.

To cluster lineages into clonotypes, we used ImmunediveRsity software (54) to create the IGH clonotypes at 100% nucleotide identity in the HCDR3. Shared IGH clonotypes within B cell subpopulations were searched at 100% of identity with R home scripts.

Differences between subpopulations and donors were assessed with the non-parametric Fisher, Wilcoxon, and Kruskal-Wallis tests. Only statistically significant differences present independently in 3 of 3 donors were considered for each of the parameters evaluated.

## Results

### IgM^hi^ and IgM^lo^ B Cells Differ Phenotypically

We have previously used GFP-VLP to identify and study human RV-B cells (4, 7, 8). We found that RV-IgM^+^IgD^+^CD27^+^ B cells can be grouped into two subpopulations: one with high levels of IgM and low levels of IgD (IgM^hi^) and another with low expression of IgM and high expression of IgD (IgM^lo^). Here, using an improved staining combination of antibodies (Supplementary Figures 1A,B), we found that although RV-B cells are enriched in both subpopulations, IgM^hi^ and IgM^lo^ B cells, most RV-IgM B cells are IgM^hi^ (twofold change) (Supplementary Figure 1C). Also, IgM^hi^ B cells made up roughly two-thirds and IgM^lo^ B cells one-third of total IgM^+^IgD^+^CD27^+^ B cells (Supplementary Figure 1C). These results confirmed our previous findings (7, 8) and led us to hypothesize that RV specificity discriminates two human circulating IgM B cells in total IgM^+^IgD^+^CD27^+^ B cells of healthy donors. After excluding plamablasts, a median of 3.9 and 6.62% of CD19 B cells are IgM^lo^ and IgM^hi^ B cells, respectively (Supplementary Figure 1D). Moreover, we noted these B cell subpopulations were notoriously distinct in a patient with a 22q11.2 distal deletion syndrome associated with a p. Ala7dup variant in the *MAPK1* gene (Supplementary Figure 2) (55). Here, we sought to further characterize these two subpopulations of IgM B cells.

**FIGURE 1:**
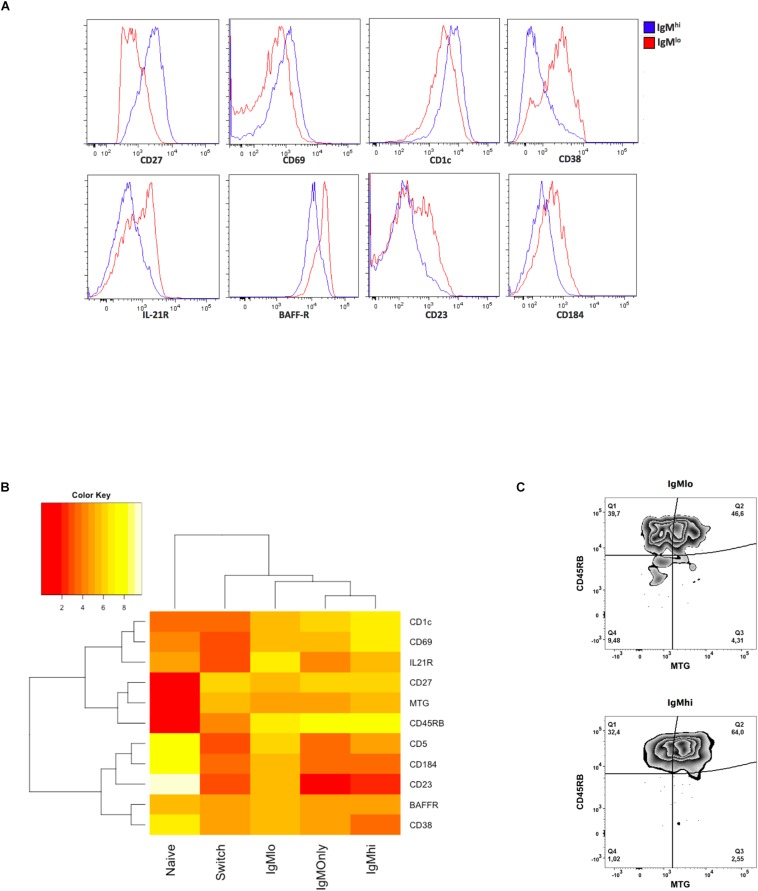
Phenotypic profile of B cells subsets. Twenty-six markers were evaluated by flow cytometry on the different B cells subsets (complete results are presented in Supplementary Table 1). **(A)** Representative histograms of selected markers for which a statistically significant difference was observed with the Kruskal-Wallis and Wilcoxon signed-rank test, *p* < 0.05, between IgM^hi^ (blue) and IgM^lo^ (red) B cells. **(B)** Unsupervised clustering analysis of the median normalized iMFI from markers that were statistically different between IgM^hi^ and IgM^lo^ B cells. *n* = 5–18. To normalize the iMFI, raw values were square root transformed. Each transformed value was then multiplied by five and divided by the average of the transformed values in all five B cells subsets for each marker. **(C)** Representative plot of the joint expression of CD45RB (MEM55) vs. MTG in one volunteer, *n* = 7.

Employing a similar but not identical staining strategy, other investigators determined that IgM^lo^ B cells differ from IgM^hi^ B cells by the high expression of several markers (56). To compare the phenotype of IgM^hi^ and IgM^lo^ B cells, we evaluated 26 markers previously evaluated by us (36) and others (27, 37, 56) in PBMCs, and used naïve B cells, IgM only Bmem cells, and class-switched Bmem cells for comparison. Eleven markers (excluding IgM and IgD) showed statistically significant differences between IgM^hi^ and IgM^lo^ subsets (Supplementary Table 1 and Figures 1A,B). Comparatively higher expression of CD27, CD69, CD1c, CD45RB (MEM55), and MTG was observed in IgM^hi^ B cells, and higher expression of CD38, IL21R, BAFF-R, CD23, CD184, and CD5 in IgM^lo^ B cells (Supplementary Table 1 and Figure 1A). An unsupervised clustering analysis including the iMFI of the 11 markers that differ between both subpopulations separated naïve B cells from all other subsets and group all the IgM expressing B cells separately from class-switched Bmem cells (Figure 1B). However, IgM^hi^ B cells and IgM only Bmem cells formed a separate subgroup from IgM^lo^ B cells (Figure 1B). It is worth noting that IgM^lo^ B cells expressed lower levels of CD27, CD45RB, and MTG (Figure 1B). In addition, IgM^hi^ B cells were enriched in CD45RB^+^MTG^+^ B cells, while IgM^lo^ B cells presented higher frequencies of CD45RB^+^MTG^–^, CD45RB^–^MTG^+^, and CD45RB^–^MTG^–^ cells (Kruskal-Wallis *p* < 0.0001 to *p* < 0.0008, Wilcoxon test *p* = 0.01 in all cases) (Figure 1C and data not shown). Thus, IgM^lo^ and IgM^hi^ B cells differ phenotypically, with IgM^lo^ being a less differentiated B cells subset.

### IgM^hi^ and IgM^lo^ B Cells Differ Transcriptomically by the Expression of 78 Genes

**FIGURE 2:**
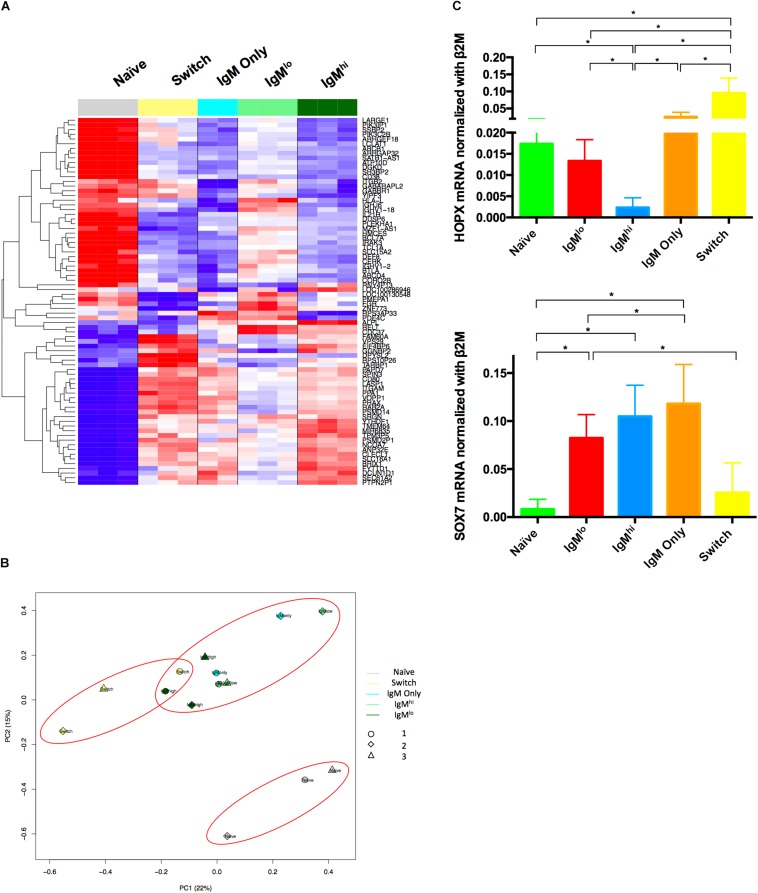
Transcriptomic profile of B cells subsets. **(A)** Clustering analysis of the 78 genes that differed in expression between IgM^hi^ and IgM^lo^ B cells subsets with an FDRq LME-ANOVA < 0.001 and a *p* < 0.01. **(B)** PCA of Brainarray renormalized transcriptomic data adjusted by donor of the five B cells subsets performed as described in materials and methods. *n* = 3 for all subpopulations except for *n* = 2 for IgM only. Gene expression values are color coded, ranging from blue (low expression) to red (high expression), scaled by row, **(C)** RNA purified from sorted B cells were analyzed for the relative expression of *HOPX* and *SOX7*. n = 6. The relative expression of these genes normalized with the *B2M* gene is shown. Kruskal-Wallis and Wilcoxon test were used for evaluating differences among groups *p ≤ 0.05.

Other investigators have reported the transcriptomic profile of circulating IgM^+^IgD^+^CD27^+^ B cells purified, in most cases, with a similar sorting strategy used in this work (26, 33, 57). To compare the transcriptome of IgM^hi^ and IgM^lo^ B cells, we sort purified these B cells subsets from the same individual, three blood donors in total, and the RNA extracted from the B cells was analyzed with Clariom D microarrays, as described in section “Materials and Methods.” RNA extracted from sort purified naïve B cells, IgM only Bmem cells, and class-switched Bmem cells were run in parallel for comparison. The expression profiles of IgM^hi^ and IgM^lo^ B cells were similar, and only 78 genes differed between them with an FDRq LME-ANOVA < 0.001 and a *p* < 0.01 (Figure 2A and Supplementary Table 2). A clustering analysis considering the expression of these 78 genes showed that IgM^hi^ B cells were similar to IgM only and class-switched Bmem cells (B cells that have passed through germinal centers), while the gene profile of IgM^lo^ B cells differed from those of these three subsets and was somewhat closer to the gene profile of naïve B cells (Figure 2A). A PCA of 39,337 Brainarray renormalized genes showed that IgM^hi^ and IgM^lo^ B cells clustered with IgM only Bmem cells and diverged from naïve B cells and class-switched Bmem cells (Figure 2B). However, IgM^hi^ B cells tended to be closer to class-switched Bmem cells than IgM^lo^ B cells (Figure 2B).

A gene set enrichment analysis (GSEA) revealed 159 biological processes enriched in IgM^hi^ B cells with respect to IgM^lo^ B cells with an FDR < 0.25, 10 of which corresponded to B cells related processes (data not shown). Three of these processes suggest that IgM^hi^ B cells, relative to IgM^lo^ B cells, resemble Bmem cells, because they expressed genes downregulated in naïve B cells with respect to Bmem cells (GSE12366 NAIVE VS MEMORY B CELL DN). Moreover, IgM^hi^ B cells appear to be more activated, in comparison with IgM^lo^ B cells, given that they are related to sets of genes associated with the negative regulation of B cells activation (GO NEGATIVE REGULATION OF B CELL ACTIVATION) and the negative regulation of antigen receptor signaling (GO NEGATIVE REGULATION OF ANTIGEN RECEPTOR MEDIATED SIGNALING PATHWAY).

Previously, a study reveled that MZB cells from spleen and IgM^+^IgD^+^CD27^+^ B cells from blood have a marked positive regulation of the *SOX7* transcription factor, and negative regulation of the *COCH* gene and the *TOX* and *HOPX* transcription factors, with respect to class-switched Bmem cells (26). Also, in samples from spleens of children, *SOX7* was expressed at very low levels in naïve B cells and at low levels in MZPc, while *HOPX* was expressed at intermediate levels in naïve B cells, compared with class-switched Bmem cells, and expressed at low levels in MZPc (26). We performed qRT-PCR for these genes with purified B cells of five different blood donors. As expected, class-switched Bmem cells (26), but also IgM only Bmem cells, IgM^lo^ B cells, and naïve B cells expressed more *HOPX* than IgM^hi^ B cells (Figure 2C). In contrast, the levels of *SOX7* on IgM^hi^ B cells were higher than those on naïve B cells and also on class-switched Bmem cells (although only statistically significant in the former case) (Figure 2C). Thus, the patterns of expression of *HOPX* and *SOX7* genes of IgM^hi^ B cells are analogous to those reported for MZB cells, while the patterns of expression of IgM^lo^ B cells were intermediate between those of naïve and IgM^hi^ B cells (26).

### IgM^hi^ and IgM^lo^ B Cells Differentially Proliferate, Differentiate to Antibody Secreting Cells (ASC), and Change Isotype After Stimulation With CpG and a Cocktail of Cytokines

Various investigators, including us, have previously determined that *ex vivo* stimulation with CpG, cytokines (IL-2, IL-6, and IL-10), and irradiated NIH 3T3 fibroblasts (as feeder cells) is optimal to induce proliferation and differentiation to ASC of purified B cells (4, 58). Moreover, using this stimulus, we have shown that only a subset of sort purified IgM^+^IgD^+^CD27^+^ B cells proliferate, change isotype, and differentiate to ASC (36).

**FIGURE 3:**
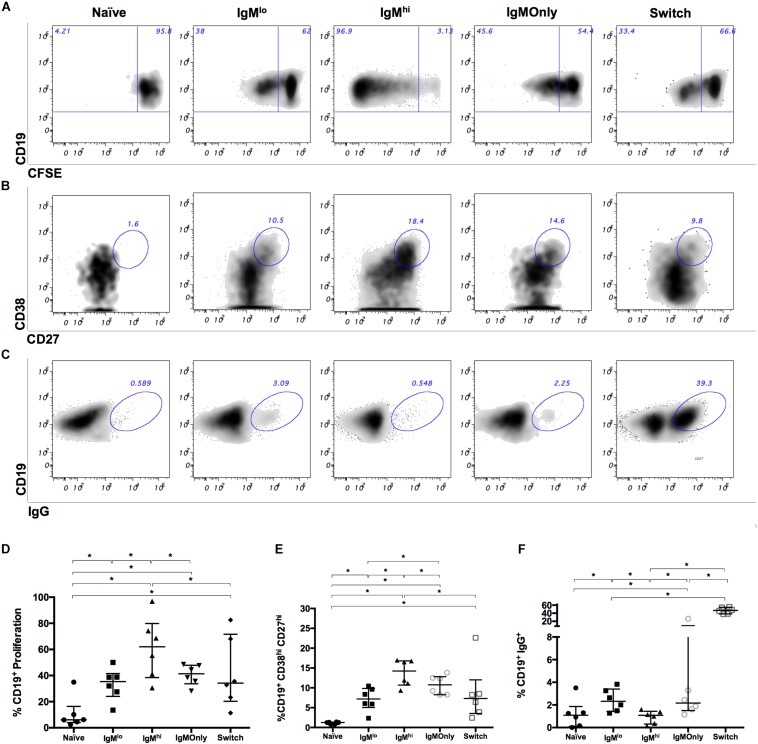
Proliferation, differentiation to ASC, and IgG expression of B cells subsets after *ex vivo* stimulation. 0.4 – 1.0 × 10^6^ sorted B cells from healthy donors were stimulated with CpG and a cocktail of cytokines (IL-2, IL-6, and IL-10) together with irradiated fibroblasts for 3 days and evaluated by flow cytometry. Representative dot plots of **(A)** CFSE- proliferating B cells, **(B)** differentiation to ASC (CD38^hi^ CD27^hi^), and **(C)** intracellular IgG expression. **(D–F)** summary of experiments presented in **(A–C)**, respectively. Wilcoxon tests were used for evaluating differences among groups. Lines and error bars denote the median and interquartile range * denotes *p* < 0.05. *n* = 6.

To evaluate the capacity of IgM^hi^ and IgM^lo^ B cells to proliferate, differentiate to ASC, and switch isotype, we stimulated sort purified B cells with CpG, cytokines (IL-2, IL-6, and IL-10), and irradiated NIH 3T3 fibroblasts. As for the gene expression experiments, we used purified naïve B cells, IgM only Bmem cells, and class-switched Bmem cells for comparison. After stimulation, higher frequencies of IgM^hi^ B cells proliferated (Figures 3A,D) and differentiated to ASCc (Figures 3B,E) compared with IgM^lo^ B cells, naïve B cells, IgM only Bmem cells, and class-switched Bmem cells. IgM^lo^ B cells proliferated (Figures 3A,D) and differentiated to ASC (Figures 3B,E) at frequencies intermediate between those of naïve B cells and IgM^hi^ B cells, and similar to class-switched and IgM only Bmem cells. Additionally, higher frequencies of IgM^lo^ B cells performed IgG class-switching in comparison with IgM^hi^ B cells; however, the observed frequencies were relatively low (Figures 3C,F) probably related to the short (3 day) stimulation time used. As expected, naïve B cells responses were undetectable. These results indicate that the IgM^hi^ and IgM^lo^ subsets proliferate, differentiate to ASC, and switch isotype differentially.

### IgM^hi^ B Cells, Compared With IgM^lo^ B Cells, Use *IGHJ6* at Lower Frequencies, Have a Higher Frequencies of Mutations in IGHV and IGKV Regions, and Differ in Their HCDR3 Properties

**FIGURE 4:**
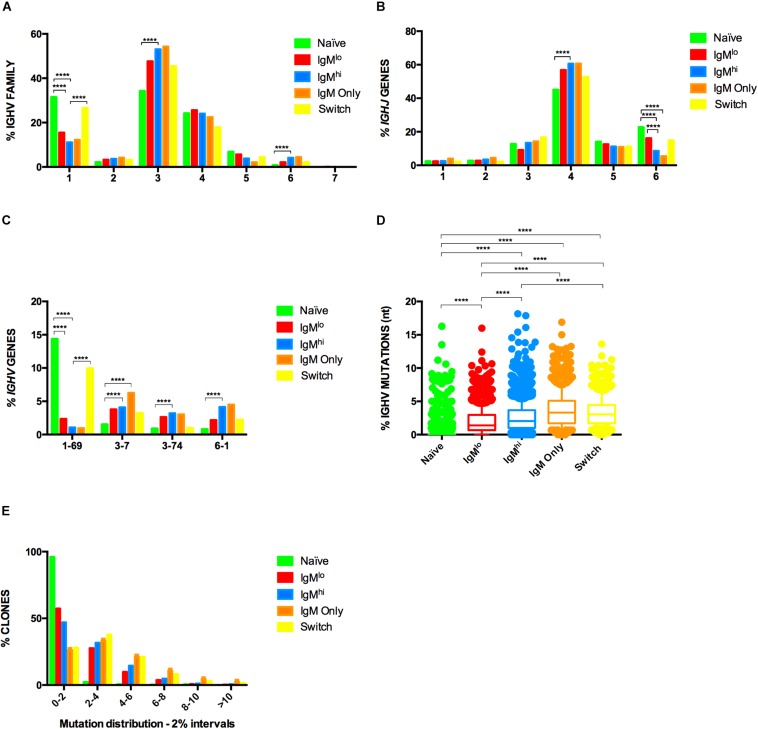
Heavy chain repertoire of B cells subsets. Frequencies of IGHV family **(A)**, *IGHJ* genes **(B)** and *IGHV* genes **(C)** used by different B cells subsets are shown. Samples from three donors were pooled and analyzed with Fisher’s test with *p*-value correction using FDR. In **(C)** shown are only the *IGHV* genes for which statistical differences were observed in all three donors studied. IGHV region mutations frequencies **(D)** were analyzed by Kruskal-Wallis test using Dunn’s multiple comparison test correction. The median and interquartile range of the three donors is shown. Only statistically significant differences observed in all three donors are shown ****p ≤ 0.00005, n = 3. **(E)** IGHV region mutations frequencies depicted as fraction of clones within six different 2% mutation intervals, from 0 to 2% (left) to >10% mutation frequency (far right).

**FIGURE 5:**
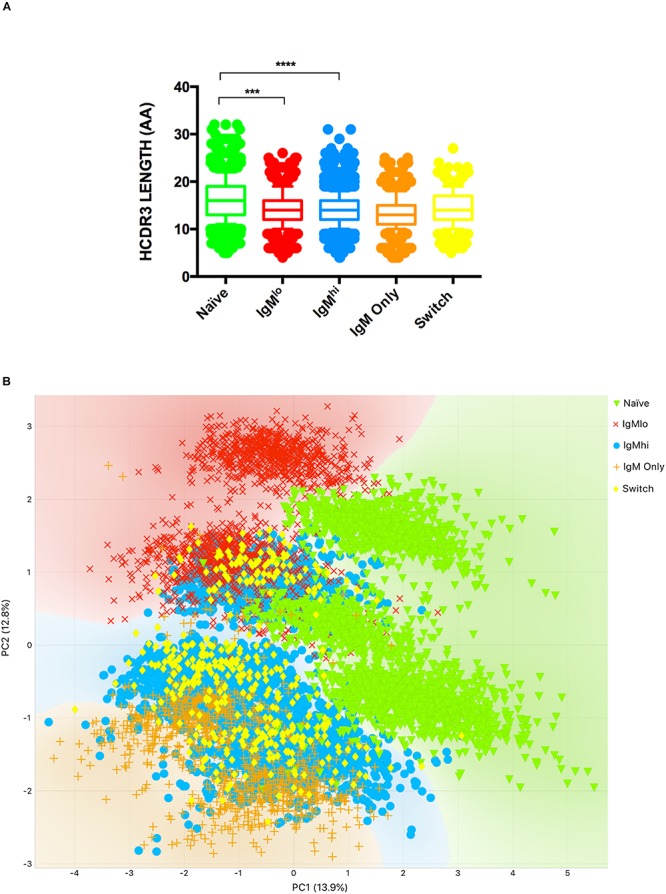
Analysis of HCDR3. The length of HCDR3 aa **(A)** and bi-plot of PCA of HCDR3 properties (performed as described in Materials and methods) **(B)** of the different B cell subsets from 3 volunteers are shown. The median and interquartile range of the three donors is shown **(A)**. Only statistically significant differences observed in all three donors are shown ***p ≤ 0.0005 and ****p ≤ 0.00005 (A), *n* = 3.

**FIGURE 6:**
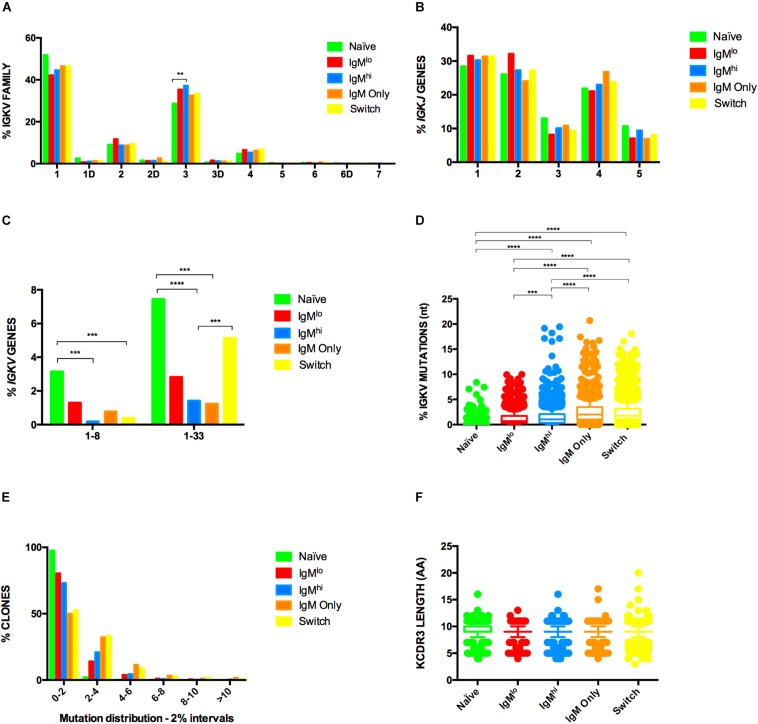
Light chain repertoire profile of B cells subsets. Frequencies of IGKV family **(A)**, *IGKJ* genes **(B)** and *IGKV* genes **(C)** used by different B cells subsets are shown. Samples from three donors were pooled and analyzed with Fisher’s test with *p*-value correction using FDR. and *IGKV* region mutations frequencies **(D)** were analyzed by Kruskal-Wallis test using Dunn’s multiple comparison test correction. The median and interquartile range of the three donors is shown. Only statistically significant differences observed in all three donors are shown. **p < 0.005, ***p < 0.0005, ****p < 0.00005, *n* = 3 IGKV region mutations frequencies depicted as fraction of clones within six different 2% mutation intervals, from 0 to 2% (left) to >10% mutation frequency (far right) **(E)**. meadian of KCDR3 length is shown **(F)**.

Characterization of the Ig gene repertoire of B cells has contributed to understanding the biology of B cells. To contrast the Ig gene repertoire of the five B cells subpopulations of interest, we sequenced the heavy (Figures 4, 5) and light (Figure 6) chain genes of purified B cells subpopulations of three adult donors. B cells subsets were characterized by usage of IGH and IGK sequences, HCDR3 structure (length, N lengths, and P lengths), mutation frequencies, and shared clonotypes.

Consistent with some previous observations (17, 22), but not others (34), IGHV family and *IGHV/IGHJ* gene usage frequencies were highly similar among B cells, with few exceptions: IgM^hi^ B cells had lower frequencies of the *IGHJ6* genes (*p* < 0.0001) compared with IgM^lo^ B cells (Figure 4B), and class-switched Bmem cells had higher expression of IGHV1 family than IgM^hi^ B cells (Figure 4A). Other differences appeared when contrasting IgM B cells subpopulations with naïve B cells: IgM^hi^ and IgM^lo^ B cells had lower levels of IGHV1 family usage (Figure 4A), and IgM^hi^ B cells had greater usage of IGHV3 and IGHV6 families (Figure 4A). Naïve B cells expressed higher frequencies of *IGHJ6* genes than IgM^hi^ and IgM only subsets, but lower frequencies of *IGHJ4* than IgM^hi^ (Figure 4B). Also compared with naïve B cells, IgM^lo^ and IgM^hi^ B cells had lower usage of *IGHV1-69* genes (Figure 4C), but IgM^hi^ had increased usage of *IGHV3-7*, *IGHV3-74*, and *IGHV6-1* genes (Figure 4C). Finally, *IGHV3-7* gene usage was significantly higher in IgM only Bmem cells compared with naïve B cells. However, a higher percentage of mutations was detected in IGHV region of IgM^hi^ B cells compared with IgM^lo^ B cells (*p* < 0.0007) (Figure 4D) and the fraction of rearrangements with 0–2% mutations for IgM^lo^ B cells was higher than IgM^hi^ B cells, while a fraction of rearrangements with more than 2% of mutations was higher in IgM^hi^ B cells (Figure 4E).As expected, naïve B cells presented the lowest frequency of mutations of the five subpopulations (Figure 4D) and 96.14% of their clones presented between 0 and 2% of mutation Figure 4E). IgM^hi^ B cells (like IgM^+^IgD^+^CD27^+^ B cells) exhibited a lower frequency of mutations than class-switched Bmem cells (Figure 4D) (17) and a higher percentage of their clones (median = 47.03%) had a range 0–2% of mutations in comparison with class-switched Bmem cells (median = 28.10%) (Figure 4E).

Additionally, in line with previous reports (17), the lengths of HCDR3 were similar among B cells subpopulations, including IgM^hi^ and IgM^lo^ B cells, but different from naïve B cells (Figure 5A). However, a PCA with the properties of HCDR3, including HCDR3 length, the lineage relative frequency, N1, and N2 length, P (P3’V, P5’D, P3’D, and P5’J) length, and percentage of identity of IGHV-IGHJ with the corresponding germline gene, grouped IgM^lo^ and IgM^hi^ B cells in two different clusters (Figure 5B). The two-component, PC1 and PC2, had a cumulative explained variance of 24.4% (13.9 and 10.5%, respectively). The differential clustering of IgM^hi^ and IgM^lo^ B cells was comparable in the 3 volunteers studied (Supplementary Figure 4).

With few exceptions, IGKV family and *IGKV/IGKJ* gene usage frequencies were highly similar among the five study populations, with frequencies corresponding to those previously reported for naïve B cells (59, 60). The *IGKV* gene families and *IGKJ* genes used by IgM^hi^ and IgM^lo^ B cells were similar (Figures 6A,B). However, compared with naïve B cells, IgM^hi^ B cells used the IGKV3 family of genes at higher frequencies and the *IGKV1-8* and *IGKV1-33* genes at lower frequencies (Figures 6A,C). Compared with class-switched Bmem cells, IgM^hi^ B cells used *IGKV1-33* genes at lower frequencies (Figure 6C). As expected, the percentage of mutations in IGKV region were lower than in the IGHV region, but, similarly to what we observed for the IGHV region, a higher percentage of mutations was found in IGKV region of IgM^hi^ B cells compared with IgM^lo^ B cells (Figure 6D). Also analogous to the IGHV region, naïve B cells presented the lowest frequency of mutations of the five subpopulations in the IGKV region (Figure 6D) and 97.6% of their clones presented between 0 and 2% of mutations (Figure 6E). IgM^hi^ B cells exhibited a lower frequency of mutations than IgM only and class-switched Bmem cells and, like in IGH, higher percentage of their clones (median = 73.09%) had a range 0–2% of mutations in comparison with class-switched Bmem cells (median = 28.10%) (Figures 6D,E). The lengths of KCDR3 were similar among all populations of B cells (Figure 6F).

In summary, assessment of the Ig gene repertoire of IgM^hi^ and IgM^lo^ B cells showed that IgM^hi^ B cells had higher frequencies of mutations in IGHV/IGKV regions, used *IGHJ6* genes at lower frequencies, and differ in their HCDR3 properties.

### Clonotypes Shared by IgM^hi^ and IgM^lo^ B Cells

To determine if IgM^hi^ and IgM^lo^ B cells were clonally related, we compared the frequency of B cells clonotypes they shared between themselves or with other B cells subpopulations, except for class-switched Bmem cells since the low number of clonotypes identified in this subset (164, 101, and 137 in donors 1, 2, and 3, respectively) limited this analysis (Table 1). A clonotype was defined as group of sequences that shared 100% of identity in nucleotides of their HCDR3 and the same assignment of *IGHV* and *IGHJ* genes reported by IMGT/HighVQuest. Considering clonotypes defined by IGH, the four subsets of B cells shared few (less than 6%) of clonotypes analyzed separately by donor (Supplementary Table 3) and when B cells from the three donors were grouped (Table 1). IgM only Bmem cells had the highest frequency of shared clones (5.78%) followed by IgM^hi^ (2.39%), IgM^lo^ (1.76%), and naïve B cells (0.04%) (Table 1). IgM^lo^ B cells shared with IgM^hi^ B cells 1.49% of clonotypes, while IgM^hi^ B cells shared 1.65% of clonotypes with IgM only Bmem cells. Interestingly, the great majority of the shared clonotypes detected in IgM only Bmem cells were almost exclusively shared with the IgM^hi^ B cells (5.39%) (Table 1 and Supplementary Table 3). No common clonotypes were identified between naïve B cells and any of the IgM B cells, except for donor number three who presented one shared clonotype between naïve B cells and IgM^hi^ B cells (0.04%) (Table 1 and Supplementary Table 3). Similar results were obtained using 97% of identity in the sequence of nucleotides of HCDR3 to define a clonotype (data not shown). In summary, IgM^hi^ B cells shared most of their shared IGH defined clonotypes with IgM only Bmem cells, and IgM^lo^ B cells shared most of their shared IGH clonotypes with IgM^hi^ B cells.

**TABLE 1:** Frequencies and percentages of clonotypes in IGH.

		**Percentage of shared clonotypes**			
**Group**	**Number of shared**	**Naïve**	**IgM^lo^**	**IgM^hi^**	**IgM**
	**clonotypes**				**Only**
IgM^hi^ Naïve	1	0.04		0.03	
IgM^lo^ IgM only IgM^hi^	2		0.14	0.06	0.20
IgM^lo^ IgM^hi^	22		**1.49**	0.66	
IgM^lo^ IgM only	2		0.14		0.20
IgM Only IgM^hi^	55			**1.65**	**5.39**
Total of shared clonotypes (%)	166	1 (0.04)	26 (1.76)	80 (2.39)	59 (5.78)
Total of unique clonotypes (%)	8447	2775 (99.96)	1449 (98.24)	3262 (97.61)	961 (94.22)
Total of clonotypes	8613	2776	1475	3342	1020

*Numbers and percentages of IGH defined clonotypes shared between B cells subsets. Clonally related sequences were identified based on 100% HCDR3 nucleotide identity, and the same usage of *IGHV* and *IGHJ* genes. Data from the three donors were pooled and total, unique, and shared clonotypes are shown for each subset. Values above 1% are shown in bold.*

## Discussion

In this work, we compared circulating IgM^hi^ and IgM^lo^ B cells. IgM^hi^ B cells share some phenotypic markers and expression of *HOPX* and *SOX7* transcription factors with MZB cells previously reported by others. They have similarities in the gene expression profile with germinal center-experienced Bmem cells (IgM only and class-switched Bmem cells), and differ from IgM^lo^ B cells in the expression of 78 genes. They have higher frequencies of mutations in IGHV/IGKV regions than IgM^lo^ Bc, share most of their shared IGH defined clonotypes with IgM only Bmem cells, and proliferate and differentiate to ASC after stimulation with a polyclonal stimulus (CpG/cytokines) at highest proportions. IgM^lo^ B cells are phenotypically closer to naïve B cells considering the low expression of CD27, CD45RB, and MTG, but present markers of Bmem cells. The patterns of expression of *HOPX* and *SOX7* genes of IgM^lo^ B cells were intermediate between those of naïve B cells and IgM^hi^ B cells. They have more significant usage of the *IGHJ6* gene, compared with IgM^hi^ B cells, share most of their shared IGHV clonotypes with IgM^hi^ B cells, proliferate and differentiate to ASC at frequencies intermediate between those of naïve B cells and IgM^hi^ B cells, and perform IgG class-switching at greater frequencies. Althogether, these results support the notion that differential expression of IgM and IgD discriminates two subpopulations of human circulating IgM^+^IgD^+^CD27^+^ B cells, with the IgM^hi^ B cells having similarities with previously described MZB cells that entered germinal centers and with the IgM^lo^ B cells being the least differentiated amongst the IgM^+^CD27^+^ subsets. In agreement with this hypothesis, we have identified a patient with 22q11 distal deletion syndrome in whom separation of these B cells subsets is notably distinct and seems to have a relative increase in IgM^lo^ B cells (Supplementary Figure 2). This patient has a chronic infection with herpes zoster virus and an increase in circulating plasmablasts (21.8% of CD19 B cells) (Supplementary Figure 2), suggesting that ongoing immune responses may favor the presence of IgM^lo^ B cells (55).

Our hypothesis that IgM^hi^ and IgM^lo^ B cells are two distinctive subsets originated with the characterization of RV-B cells (4, 7). We have confirmed here that RV-B cells are particularly enriched in the IgM^hi^ vs. the IgM^lo^ B cells (Supplementary Figures 1A–C). While the IGHV1 family is the dominant family used by naïve RV-B cells, the IGHV3 family is dominant in both IgM and class-switched RV-Bmem cells (5, 61), which is in agreement with the significant decrease and increase we found in the respective use of IGHV1 and IGHV3 families by IgM^hi^ B cells relative to naïve B cells (Figure 4A). Since RV replicates in the intestine and given the recent proposal that intestinal MZB cells diversify their Ig repertoire in GALT to later travel, via the blood, to the spleen to fulfill their function (24), it is tempting to speculate that RV selects a subset of IgM^hi^ B cells in the intestine. In line with this hypothesis, IgM^+^IgD^+^CD27^+^ B cells from GALT had relatively high expression of CD80, compared with the same subset in spleen and tonsils (24), and CD80 is among the 78 genes that differed between both IgM subpopulations (Figure 2A) and highly expressed in IgM^hi^ B cells. Also in support of this hypothesis, a relationship has been found between IgM^+^IgD^+^CD27^+^ B cells and the number of IgA secretory plasma cells and secretory IgA in gut (62). Studies to evaluate the expression of homing receptors (63) on the different B cells subsets studied here may be useful to test this hypothesis.

Other investigators have previously studied circulating CD27^+^IgM^hi^ and IgM^lo^ B cells in humans and found that the frequency of IgM^lo^ B cells decreases with age –like MZPc (26, 56)– while that of the IgM^hi^ B cells increases with age (56), like cells entering germinal centers. These authors also reported that, compared with IgM^lo^ B cells, IgM^hi^ B cells expressed lower levels of IgD, CD21, CD23, CD38, CD69, CD40, CXCR4, and Beta 7, but higher levels of CD24, CD27, CD35, CD44, and CD74 (56). Although indirectly comparable, our results are, in general –except for CD21 and CD69–, in agreement with these findings, for the markers evaluated in both studies (Figure 1 and Supplementary Table 1). In humans, MZB cells have been phenotypically defined as IgM^hi^IgD^lo^CD1c^+^CD21^hi^CD23^–^CD27^+^ (13, 16, 21, 23). We found that all these markers, excluding CD21, were differentially expressed between IgM^hi^ and IgM^lo^ B cells (Figure 1 and Supplementary Table 1), suggesting that IgM^hi^ B cells are phenotypically related to MZB cells. High expression of CD21 has been typically described on MZB cells, but was similarly expressed on circulating IgM^hi^ or IgM^lo^ B cells in our previous work (7) and the present report (Supplementary Table 1). High expression of CD21 was previously reported in MZB cells of the spleen but to a lesser extent in circulating IgD^+^IgM^+^ B cells (16, 26). Thus, circulating IgD^+^IgM^+^CD27^+^ B cells may differ in their expression of CD21 vis-à-vis spleen MZB cells. Human spleen MZB cells present a pre-activated state (23). Since IgM^hi^ B cells express significantly higher CD69 (consider a marker of early activation) than IgM^lo^ B cells (Figure 1), and the GSEA results show that IgM^hi^ B cells are related to sets of genes associated with the negative regulation of B cells activation (data not shown), it is possible that, like MZB cells, circulating IgM^hi^ B cells have this pre-activated state. An unsupervised clustering analysis including the iMFI of the 11 markers that differ between both subpopulations grouped IgM^hi^ B cells with IgM only Bmem cells that are germinal center-experienced Bmem cells (Figure 1B).

IgM^lo^ B cells, on the other hand, expressed higher levels of BAFF-R, IL-21-R, CD23, CD38, and CD184 (CXCR4) than IgM^hi^ B cells (Figure 1 and Supplementary Table 1). The expression of these five markers was comparable to naïve B cells (Supplementary Table 1), indicating that they are phenotypically related. However, in addition to CD27, CD45RB (MEM55), and MTG, expression of CD95 differentiated IgM^lo^ B cells from naïve B cells, denoting that they are distinct B cells subsets (Supplementary Table 1). CD45RB is over-expressed in the late stages of B cells differentiation (27) and the activity of ABCB1, determined by the capacity of B cells to incorporate MTG, is lost in B cells as they proliferate and differentiate (37). Interestingly, IgM^lo^ B cells expressed lower levels of CD45RB and MTG in comparison with IgM^hi^ B cells (Figure 1 and Supplementary Table 1) and IgM only Bmem cells, probable reflecting that within IgM^+^CD27^+^ subsets IgM^lo^ may present a more primitive stage of differentiation.

MZPc in spleens from children have been shown to express MTG^±^CD45RB^+^CD27^–^IgM^hi^IgD^hi^CD21^+^CD1c^*int*^CD24^*int*^ CD38^*int*^ (26). Similarly, IgM^lo^ B cells are MTG^±^CD45RB^+^ CD27^+^IgM^lo^IgD^hi^CD21^+^CD1c^*int*^CD24^hi^CD38^hi^ (Figure 1 and Supplementary Table 1). Although to our knowledge an extensive phenotype of MZPc in blood of adults has not been reported, the phenotype of IgM^lo^ B cells in blood seems compatible with the expected phenotype of these MZPc since their phenotype is somewhat similar to MZPc of children’s spleen, with the important exception of them being CD27^+^.

Our transcriptomic results showing that IgM^hi^ B cells, IgM^lo^ B cells, and IgM only Bmem cells cluster together (Figure 2B) are in agreement with those that found similarities between IgM only Bmem cells and IgM^+^IgD^+^CD27^+^ B cells (33, 64). Thus, all IgM B cells expressing CD27^+^ B cells share important gene expression patterns (33, 64). However, we found 78 genes differentially expressed on IgM^hi^ and IgM^lo^ B cells (Figure 2A), indicating that transcriptomic differences do occur between these subsets. Clustering of the different B cells subpopulations studied based on these 78 genes showed that IgM^hi^ B cells resemble IgM only and class-switched Bmem cells (Figure 2A and GSEA data not shown), supporting the notion that they enter germinal centers (24). However, expression of 9 (*ABCB1*, *BTLA*, *CERK*, *DGKD*, *GABBR1*, *MEGF6*, *SATB1-AS1*, *TCL1A*, and *CLECL1*) of those 78 genes in IgM^hi^ B cells were concordant with the relative expression of spleen MZB cells with respect to follicular naïve B cells of healthy adults (23) (Figure 2A and data not shown), suggesting that IgM^hi^ B cells may have some transcriptomic similarities with spleen MZB cells. In agreement with this finding, IgM^hi^ B cells had high gene expression of *SOX7* and low gene expression of *HOPX* (Figure 2C), which have been previously noted to differentiate spleen MZB cells and circulating IgM^+^IgD^+^CD27^+^ B cells from class-switched Bmem cells in human samples (23, 26). Thus, transcriptomically, IgM^hi^ B cells resemble both cells that have passed through germinal centers and MZB cells.

The transcriptomic analysis also evidenced similarities between IgM^lo^ B cells and naïve B cells (Figure 2A). Since MZPc are transcriptionally closer to naïve than to MZB cells (24, 26), the latter results are compatible with IgM^lo^ being less differentiated. Four of the genes coding for surface markers expressed differently between MZB cells and MZPc from spleens of children (26) were among the 78 that concordantly differed between IgM^hi^ and IgM^lo^ B cells: higher expression on IgM^lo^ B cells of *ABCB1*, *BTLA*, and *IL21R*, but lower expression of *CD80* (Figure 2A). Besides, *SOX7* and *HOPX* genes were expressed by IgM^lo^ B cells at intermediate levels between levels of naïve B cells and MZB cells. These results also suggest that IgM^lo^ B cells may be less differentiated than IgM^hi^ B cells.

We previously reported that circulating CD27^+^IgM^+^ B cells from healthy adults proliferate and differentiate to ASC after stimulation with CpG/cytokine cocktail to a greater extent than class-switched Bmem cells (36). However, in that study IgM only Bmem cells were included among the IgM Bmem cells. Here, we divided the IgM B cells population into three subsets (IgM^lo^ B cells, IgM^hi^ B cells, and IgM only Bmem cells, Supplementary Figures 1A,B) and established that more significant percentages of IgM^hi^ B cells proliferated (Figures 3A,D) and differentiated to ASC (Figures 3B,E) after treatment with this same stimulus, compared with IgM^lo^ B cells, IgM only Bmem cells, and class-switched Bmem cells. In these experiments, IgM^lo^ B cells responded at frequencies intermediate between those of naïve B cells and IgM^hi^ B cells, and similarly to class-switched and IgM only Bmem cells. Thus, of the three subpopulations of IgM B cells studied, IgM^hi^ B cells subset, is the most enriched in cells capable of proliferating in response to the CpG/cytokine cocktail. We have previously determined that after stimulation with the CpG/cytokine stimulus we have used in the present experiments (Figure 3), the generation of ASC from IgM B cells purified by sorting based on the expression of CD27 and the lack of expression of IgG and IgA was comparable to that of IgM B cells sorted based on the expression of CD27, IgM, and IgD (7). This result suggests that the CpG and cytokines used for stimulation, and not the anti-IgM and IgD used for the sorting of the cells, are the dominant stimulus in this type of assays. Thus, given that in our experimental conditions the CpG and cytokines (that resembles an innate stimulus) is the principal stimulus, the differences in proliferation, differentiation to ASC, and isotype switch of IgM^hi^ and IgM^lo^ B cells (Figure 3) are probably due to the fact that IgM^hi^ B cells are related to MZB cells, for which a response against CpG is expected to be higher (21).

Previous analyses of the Ig gene repertoire of IgM^+^IgD^+^CD27^+^ B cells delivered inconsistent results (17, 22). One study found that circulating IgM^+^IgD^+^CD27^+^ B cells use similar *IGHV* gene segments as IgM only and class-switched Bmem cells, and a strong clonal relationship of this population with IgM only Bmem cells was identified (17). In contrast, other studies established differences in the use of *IGHV* gene segments among these B cells populations: circulating IgM^+^IgD^+^CD27^+^ B cells had increased usage of *IGHV4* and *IGHJ6* genes in comparison with IgM only Bmem cells (22), and *IGHV3* genes in comparison with class-switched Bmem cells (34), and strong clonal relationships of this population with class-switched Bmem cells were undetected in both studies. In general, we found similarities between IgM^hi^ and IgM^lo^ B cells in the use of families and genes for both IGH and IGK, except for *IGHJ6* genes, used at higher frequencies by IgM^lo^ B cells (Figures 4A–C, 6A–C). However, IgM^hi^ B cells, unlike IgM^lo^ B cells, used IGHV3 family and *IGHJ4* genes at higher frequencies than naïve B cells (Figures 4A,B). We also found IgM^hi^ B cells used IGHV1 family genes (and the *IGHV1-69* gene) at lower frequencies than class-switched Bmem cells (Figures 4A,C). In agreement with our findings (Figure 4B), naïve B cells have been reported to use higher frequencies of *IGHJ6* and lower frequencies of *IGHJ4* genes relative to Bmem cells (34, 65), again supporting the relationship between IgM^lo^ and naïve B cells.

To our knowledge, this is the first report contrasting the repertoire of IGK families and genes of human circulating naïve B cells with other B cells subsets. We found a relatively homogeneous use of *IGKV* and *IGKJ* genes in the five study populations, with frequencies corresponding to those previously reported for naïve B cells (59, 60). However, IgM^hi^ B cells used *IGKV1-33* genes at lower frequencies than class-switched Bmem cells and naïve B cells (Figure 6C).

In agreement with our results (Figures 4D,E, 6D,E), the frequencies of mutations in IGHV and IGK regions of human circulating class-switched and IgM only Bmem cells have been reported to be higher than that of IgM^+^IgD^+^CD27^+^ B cells (17, 22). Analogous to what was initially reported by Küppers *et.*, *al* (66) and confirmed recently (67), the percentage of mutations in the IGKV region was lower compared with that of the IGHV region (Figures 4, 6). Furthermore, the percentages of mutations in IGHV and IGKV regions of IgM^lo^ B cells were lower than the ones of IgM^hi^ B cells (Figures 4D,E, 6D,E), which is coherent with IgM^lo^ B cells being in a developmental stage closer to naïve B cells. Similarly, in mice, IgM^hi^ B cells identified with a tetramer specific for the *Plasmodium* MSP1 protein had a higher percentage of mutations than IgM^lo^ B cells (68). Ig genes of MZPc of spleens of children were mostly unmutated (26), and, although not directly comparable, our results suggest that IgM^lo^ B cells have frequencies of mutations in IGHV regions at intermediate levels between MZPc and MZB cells (Figure 4D). Once more, this result indicates that IgM^lo^ B cells may be less differentiated than IgM^hi^ B cells.

While we did not observe differences in the length of both HCDR3 (Figure 5A) and KCDR3 (Figure 6F) between IgM^lo^ and IgM^hi^ B cells, a PCA analysis of the properties of HCDR3 showed that IgM^lo^ and IgM^hi^ B cells are differentially clustered (Figure 5B). This result further supports our hypothesis that IgM^lo^ cells and IgM^hi^ are two separate subpopulations of B cells and suggests that the antigens that selected them are different, and, thus, that they may have different functions.

Analysis of the IGH clones, permitted us to evaluate clonal relationships between the IgM expressing B cells (Table 1 and Supplementary Table 3). The highest percentages of clonotypes shared by IgM^lo^ B cells, IgM^hi^ B cells, and IgM only Bmem cells are with IgM^hi^ B cells, IgM only Bmem cells, and IgM^hi^ B cells, respectively (corresponding to most of the numbers shown in bold in Table 1). It is noteworthy that the percentage of clonotypes shared between the IgM^lo^ and IgM^hi^ B cells is relatively low, but support the notion that IgM^lo^ and IgM^hi^ B cells are related and consistent with IgM^lo^ B cells being less differentiated than IgM^hi^ B cells. In contrast, there seems to be a higher clonal relationships between IgM^hi^ B cells and IgM only Bmem cells (Table 1), suggesting that these two subpopulations are developmentally related.

In conclusion, we have shown that circulating IgM^hi^ and IgM^lo^ B cells differ phenotypically (Figure 1 and Supplementary Table 1), transcriptomically (Figure 2), functionally *in vitro* after stimulation with CpG and cytokines (Figure 3), genetically in their frequency of mutations in IGHV and IGKV regions, usage of the *IGHJ6* gene (Figures 4, 6), and HCDR3 properties (Figure 5), and clonotypically (Table 1). IgM^hi^ B cells have characteristics of MZB cells with features of post-germinal center Bmem cells, the frequency of which may increase with age (56), as B cells entering germinal centers. These characteristics of IgM^hi^ B cells are in agreement with the current literature concerning IgM^+^IgD^+^CD27^+^ B cells (10–13) and are consistent with the concept that their heterogeneity is generated by gradual recruitment of MZB cells into germinal center responses (35). In contrast, our findings with IgM^lo^ B cells seem novel: IgM^lo^ B cells display markers of MZPc (26, 27), the frequencies of which decrease with age (26, 56), have some clonal relationships with IgM^hi^ B cells, and appear less differentiated, suggesting that IgM^lo^ B cells may be the precursors of IgM^hi^ B cells. However, IgM^lo^ B cells may include other subsets: in mice, long-lived IgM Bmem cells with characteristics of stem cells, homologous to stem T cells have been identified (69). IgM^lo^ B cells have the lowest level of mutations of the CD27^+^ subset and greater expression of CXCR4 than other Bmem cells (Supplementary Table 1), which seems relevant, because SDF/CXCR4 are implicated in the maintenance or differentiation of stemness (70). More studies are needed to determine if IgM^lo^ B cells are heterogeneous and if some of them have features of stem Bmem cells that would make them excellent targets for vaccines (71–73).

## Data Availability Statement

The microarray and Ig genes sequences generated for this study can be found in the Gene Expression Omnibus (GEO) and NCBI-SRA repositories. The accesion numbers are GSE149089 and PRJNA625972, respectively.

## Ethics Statement

The studies involving human participants were reviewed and approved by Ethics Committee of the School of Medicine of the Pontificia Universidad Javeriana and Hospital Universitario San Ignacio. The patients/participants provided their written informed consent to participate in this study. Written informed consent was obtained from the individual(s) for the publication of any potentially identifiable images or data included in this article.

## Author Contributions

DB performed a subset of flow cytometry experiments for the phenotypic characterization of B cells subsets, transcriptomic, and Ig gene repertoire experiments, and contributed to writing the manuscript. CV characterized the phenotype, sorted B cells subsets, performed qRT-PCR experiments, and functional B cells studies. PA-R helped in the design and advised in performing the qRT-PCR experiments. EG-L performed HCDR3 PCA and intraclonal diversity analysis. JT-S and JM-B designed and advised in performing the experiments to determine the Ig gene repertoire of B cells subsets. MF and JA wrote and obtained funding for the project, directed the work, and wrote the manuscript.

## Conflict of Interest

The authors declare that the research was conducted in the absence of any commercial or financial relationships that could be construed as a potential conflict of interest.

## Footnotes

1 http://brainarray.mbni.med.umich.edu/Brainarray/Database/CustomCDF

2 https://www.bioinformatics.babraham.ac.uk/projects/fastqc/

3 http://www.imgt.org

4 https://cran.r-project.org/web/packages/Peptides
