# Supplementary material for: Differential Expression of IgM and IgD Discriminates Two Subpopulations of Human Circulating IgM+IgD+CD27+ B Cells That Differ Phenotypically, Functionally, and Genetically
Source: Front Immunol. 2020 May 6;11:736. doi: 10.3389/fimmu.2020.00736 (PMC7219516; doi:10.3389/fimmu.2020.00736)
Supplement: Supplementary file 1 [file Presentation_1.pdf]

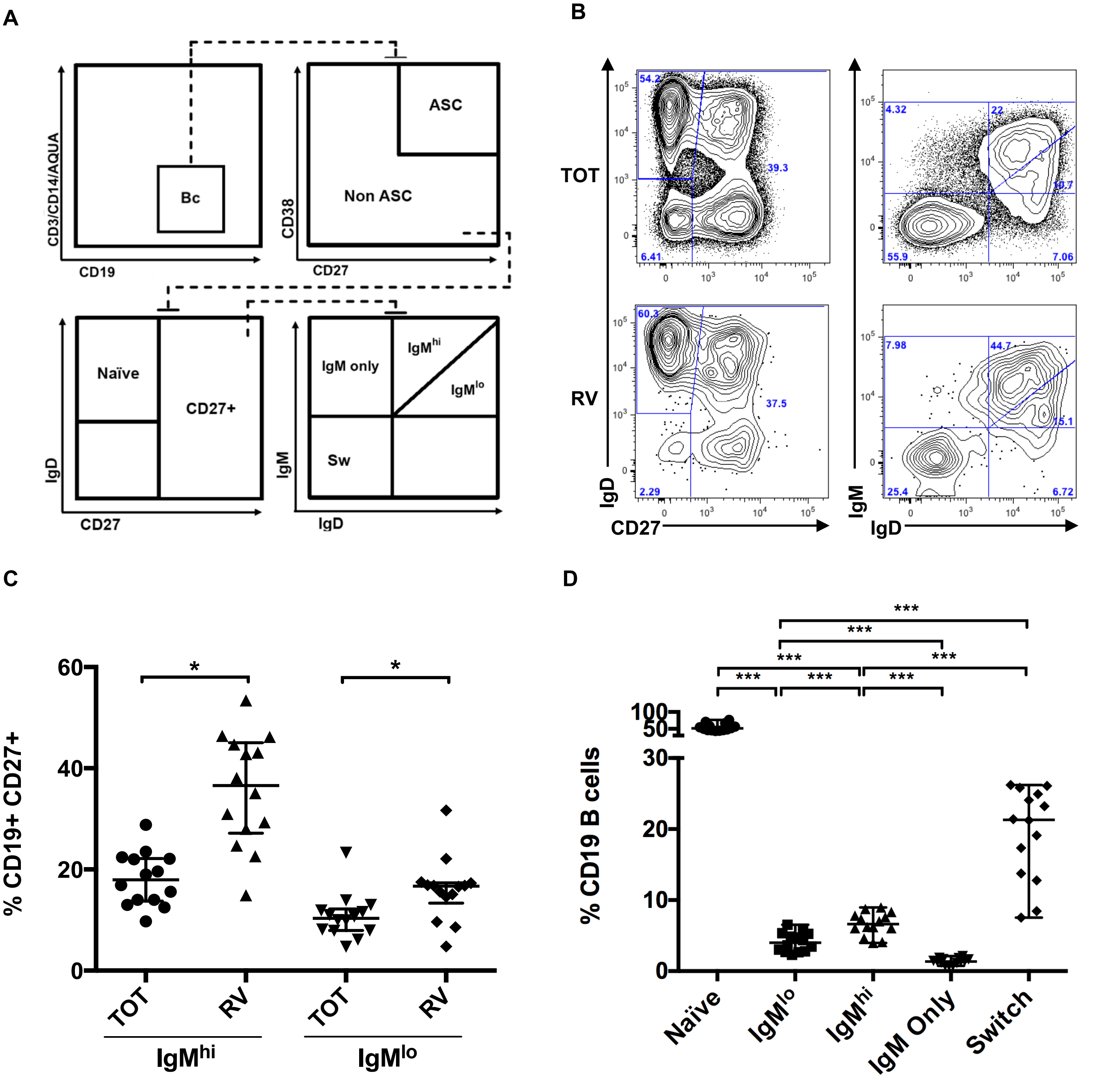

Supplementary Figure 1. RV-specific B cells were enriched in the IgM^{hi} subset. PBMCs from healthy donors were isolated and stained with GFP-VLPs, anti-CD19, anti-CD38, anti-CD27, anti-IgD, and anti-IgM. (A) Flow cytometry gating strategy. After an initial gate on live (dump channel- cells) CD19+ B cells, ASC (CD38^{hi}, CD27^{hi}) are excluded (top two graphs). Then, CD19+CD27+, total and RV-specific cells (GFP-VLP+) were gated, analyzed for the expression of IgD and IgM to define class-switched (Sw) Bmem cells, IgM only Bmem cells, and IgM^{hi} and IgM^{lo} B cells. (B) A representative example of dot plots for total and RV-B cells is presented. (C) Shown are the percentages of CD19^{+} CD27^{+} total and RV specific IgM^{hi} and IgM^{lo} B cells. (D). Percentages of Naïve, IgM^{lo}, IgM^{hi}, IgM Only and class-switched B cells are shown. Wilcoxon tests were used for evaluating differences among groups. Lines and error bars denote the median and interquartile range * denotes $p<0.05$. n=14.

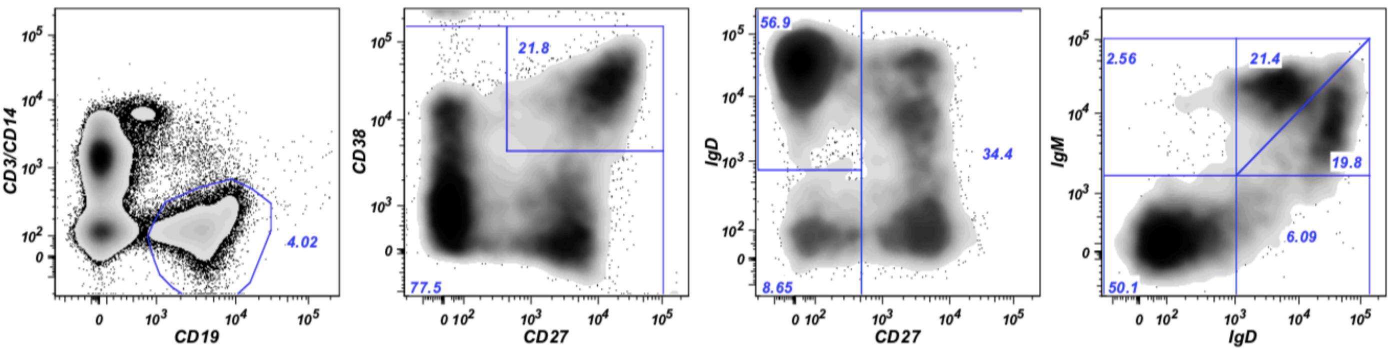

Supplementary Figure 2. IgM^{hi} and IgM^{lo} B cells were identified in a patient with 22q11.2 distal deletion syndrome associated with a p. Ala7dup variant in the *MAPK1* gene. PBMCs from the patient were isolated and stained with anti-CD19, anti-CD38, anti-CD27, anti-IgD, and anti-IgM and analyzed as in Supplementary Fig. 1.

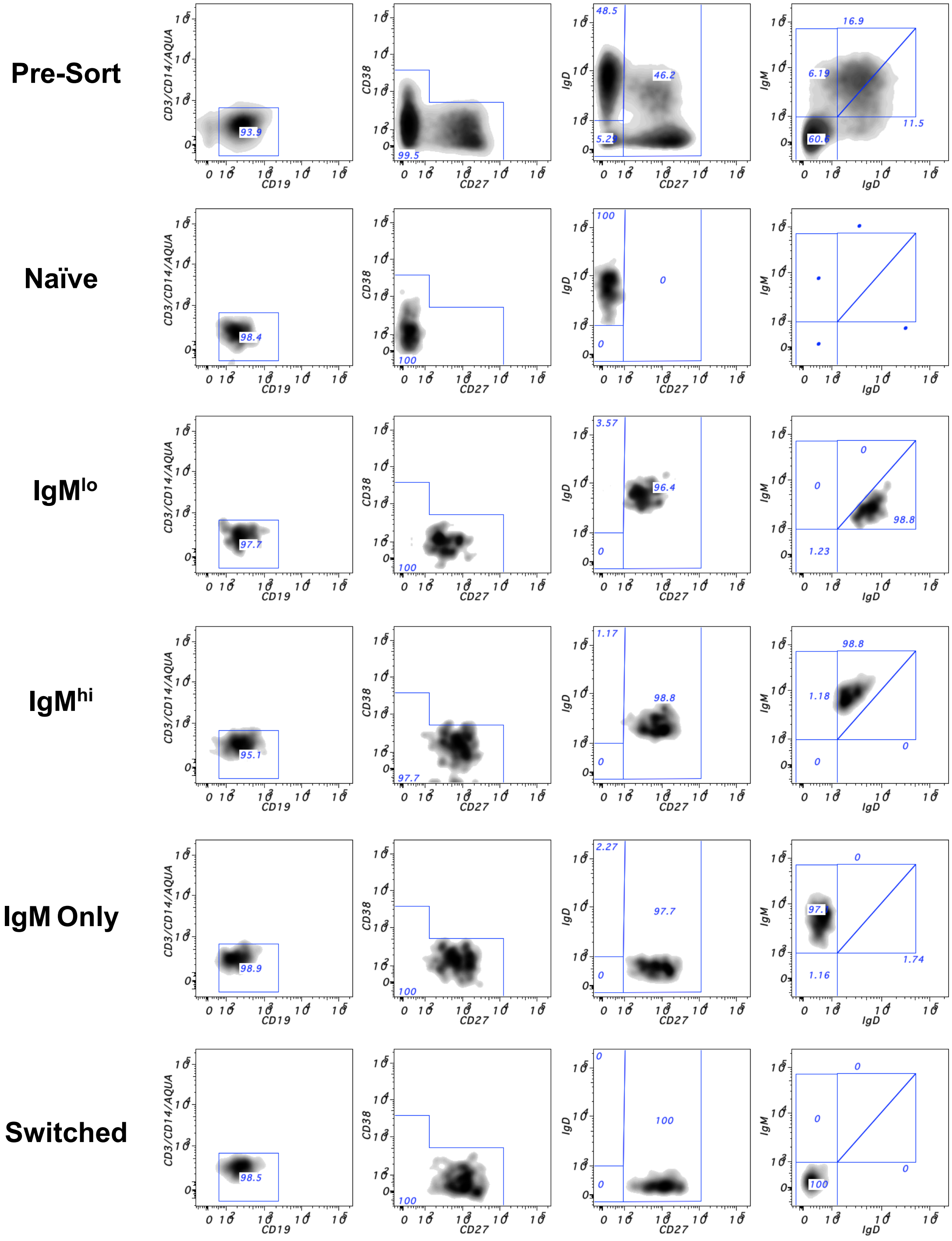

Supplementary Figure 3. Purity of B cells subsets obtained after the sorting process. Representative image of the purities obtained after the sorting process from one volunteer. The percentage of cells obtained from top to bottom of the pre-sort is shown, naïve, IgM^{lo}, IgM^{hi} B cells, IgM Only and switched Bmem cells.

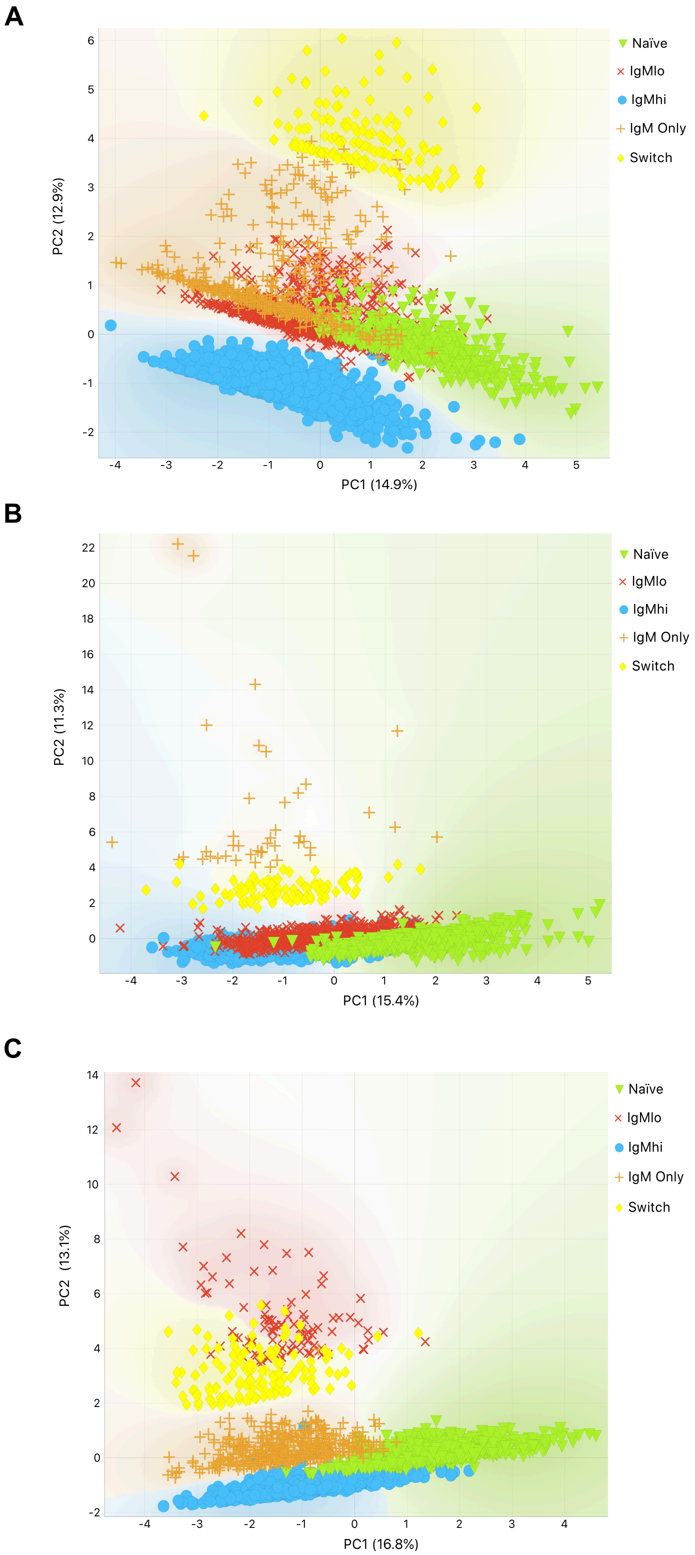

Supplementary Figure 4. PCA of HCDR-3 properties. The HCDR-3 length, the relative frequency (number of reads per lineage/total reads in lineages ≥ 5 reads), N1 and N2 length, P (P3'V, P5'D, P3'D, and P5'J) length, and % identity of IGHV-IGHJ with the corresponding germline gene were used as input to PCA. Donor 1 (A), donor 2 (B), donor 3 (C).

Supplementary Table 1. iMFI of markers evaluated on five B cell subsets. iMFI of markers evaluated on five B cell subsets. Twenty-six markers were evaluated on five B cell subsets. Median and standard deviation of iMFI from these molecules are shown. n=5-18*.

Supplementary Table 1. iMFI of markers evaluated on five B cell subsets

	**Subsets**					**K-W**	**Wilcoxon Signed Rank Test**				
**Marker**	**Naïve**	**IgM$_{lo}$**	**IgM$_{hi}$**	**IgM Only**	**Class-switched**		**IgM$_{hi}$ *vs.* IgM$_{lo}$**	**IgM$_{hi}$ *vs.* IgM Only**	**IgM$_{hi}$ *vs.* class-switched**	**IgM$_{lo}$ *vs.* class-switched**	**IgM Only *vs.* class-switched**
**BAFFR n=16**	1300913±347594	1523103±562629	1047876±352963	906105±253938	1038235±334462	***	***	**	ns	***	*
**CD1c n=5**	98261±27952	318128±53030	460194±85821	324003±81872	107447±23555	***	*	*	*	*	*
**CD184 n=7**	52980±16924	25737±9931	15752±9932	12222±8282	14114±9345	**	*	*	ns	**	*
**CD69 n=5**	19228±4151	29572±6536	41972±6930	30204±5733	10485±2533	***	*	*	*	*	*
**CD38 n=15**	136343±55849	85405±54027	37737±18734	70833±27772	66633±20303	***	***	***	***	ns	ns
**CD23 n=5**	50968±22038	18104±10158	3959±3423	1392±526	5276±1631	***	*	*	ns	*	*
**IL21R n=10**	8446±6733	13959±8739	6330±3162	6621±5921	1612±712	***	**	ns	***	***	**
**IgM n=7**	137500±52737	344171±62312	937686±185567	559657±160523	0±0	***	*	*	*	*	*
**IgD n=7**	965086±311114	679914±164580	265329±46358	0.2±0	0.1±0	***	*	*	*	*	*
**CD27 n=8**	0.04±0.02	167825±53229	199975±50543	231300±57463	224463±59160	**	*	ns	*	*	ns
**CD5 n=6**	25009±13065	12524±4644	7423±2524	3304±1231	5288±3546	***	*	*	ns	ns	ns
**CD95 n=7**	6717±2501	93924±30259	103618±41575	203023±48111	340503±132014	***	ns	**	**	**	**
**CD11b n=5**	1913±2560	10951±9966	11534±5554	11181±6672	14599±9604	*	ns	ns	ns	*	ns
**CD11c n=5**	4188±2700	25746±15117	24253±12614	34122±18100	28480±14176	**	ns	*	ns	ns	*
**CD45RO n=5**	895±542	5176±4033	1766±1254	9244±7977	3202±1634	*	ns	ns	*	ns	ns
**CD45RB n=7**	19663±12246	1261308±458944	1686824±559132	1525690±507795	652607±196024	****	*	*	*	*	*
**MTG n=7**	26614±27647	313552±235052	435508±313658	494699±365408	522988±412747	**	*	ns	ns	*	*
**CD40 n=18**	912482±464207	1060990±620135	858488±481876	755376±392154	858003±448385	ns	***	***	ns	**	**
**CD24 n=7**	151715±135412	550646±464592	786362±654270	682848±533819	273912±255011	ns	*	*	**	**	**
**CD73 n=7**	169714±119082	104408±83702	47682±39732	117710±164389	203237±182882	ns	*	ns	*	ns	ns
**CD122 n=6**	2854±3733	8577±7975	7487±8944	4842±6508	2848±3159	ns	ns	*	*	*	ns
**PD-1 n=5**	1059±631	6147±5424	4766±5356	2573±2095	1559±870	ns	ns	*	*	*	ns
**TLR9 I n=5**	71682±22975	101789±34778	104046±34519	105925±34978	98921±34866	ns	ns	ns	*	ns	*
**TLR9 n=6**	1247±1619	5900±6760	3659±3842	2120±2203	1350±1396	ns	ns	*	*	*	*
**CD21 n=6**	403062±498991	564116±774610	363595±443890	314576±374458	322380±373928	ns	*	*	ns	*	ns
**CD43 n=5**	3383±4710	24654±24871	10264±14500	89099±185536	77186±138146	ns	ns	*	*	ns	ns

*Kruskal-Wallis and Wilcoxon signed-rank tests were used for evaluating differences among Bc subsets: $p \geq 0.05$ (ns: not significant), $p \leq 0.05$ (*), $P \leq 0.01$ (**), $p \leq 0.001$ (***), $p \leq 0.0001$ (****)

Supplementary Table 2. List of differentially expressed genes between IgM$_{hi}$ and IgM$_{lo}$ B cells. Entrez Gene ID, symbol, and a short description of 78 differentially expressed genes between IgM$_{hi}$ and IgM$_{lo}$ B cells are shown*.

Supplementary Table 2. List of differentially expressed genes between IgM$_{hi}$ and IgM$_{lo}$ B cells

Entrez Gene ID	Symbol	Description	log2 (expression)													
			Naïve			Switch			IgM only		IgMlo			IgMhi		
			1	2	3	1	2	3	1	3	1	2	3	1	2	3
9215	LARGE1	LARGE xylosyl- and glucuronyltransferase 1	6,31	6,46	6,39	5,39	5,34	5,34	4,78	4,73	5,18	5,49	5,48	4,54	4,73	4,90
113791	PIK3IP1	phosphoinositide-3-kinase interacting protein 1	7,31	7,07	8,25	6,88	6,16	7,51	6,26	6,02	6,67	6,32	7,50	6,27	5,97	7,07
23635	SSBP2	single stranded DNA binding protein 2	7,35	7,38	7,01	6,32	6,45	6,24	5,36	5,11	5,78	6,08	6,32	5,13	5,52	5,71
5287	PIK3C2B	phosphatidylinositol-4-phosphate 3-kinase catalytic subunit type 2 beta	6,94	7,03	6,96	6,04	6,26	5,98	5,47	5,44	5,98	5,93	6,04	5,41	5,48	5,59
23370	ARHGEF18	Rho/Rac guanine nucleotide exchange factor 18	7,20	7,48	7,37	6,95	7,15	7,03	6,71	6,81	6,88	7,00	6,97	6,64	6,83	6,75
253558	LCLAT1	lysocardiolipin acyltransferase 1	5,36	5,02	4,90	4,86	4,78	4,48	4,70	4,50	4,99	4,65	4,63	4,87	4,52	4,52
5243	ABCB1	ATP binding cassette subfamily B member 1	3,78	4,11	3,73	2,19	2,39	2,37	2,08	2,12	2,43	2,43	2,64	2,10	2,12	2,27
9743	ARHGAP32	Rho GTPase activating protein 32	4,71	4,31	5,03	2,49	2,47	2,59	2,41	2,26	2,59	2,62	2,66	2,37	2,44	2,43
101927777	SATB1-AS1	SATB1 antisense RNA 1	4,72	4,82	4,74	3,29	3,38	3,38	3,34	3,33	3,32	3,61	3,51	2,89	3,21	3,20
57205	ATP10D	ATPase phospholipid transporting 10D (putative)	6,30	6,38	5,85	4,66	4,79	4,91	4,39	4,34	5,08	4,93	5,15	4,63	4,51	4,77
8527	DGKD	diacylglycerol kinase delta	7,25	7,44	7,18	6,46	6,51	6,23	6,02	6,19	6,41	6,51	6,35	6,15	6,28	6,03
6452	SH3BP2	SH3 domain binding protein 2	6,37	6,49	6,76	5,68	5,59	5,61	5,40	5,50	5,68	5,68	6,00	5,40	5,33	5,64
952	CD38	CD38 molecule	5,14	5,94	5,30	3,85	4,12	4,02	3,81	3,91	3,49	3,91	3,91	3,21	3,60	3,62
3689	ITGB2	integrin subunit beta 2	6,84	6,75	6,98	6,95	6,62	6,66	6,26	6,18	6,83	6,74	6,65	6,53	6,45	6,35
11345	GABARAPL2	GABA type A receptor associated protein like 2	7,10	7,12	7,18	7,15	6,98	7,09	6,56	6,41	6,93	6,89	7,08	6,74	6,64	6,84
2550	GABBR1	gamma-aminobutyric acid type B receptor subunit 1	7,09	7,39	6,83	6,96	6,94	6,72	6,59	6,67	6,86	6,91	6,60	6,62	6,70	6,40
25844	YIPF3	Yip1 domain family member 3	5,82	5,63	5,84	5,77	5,58	5,71	5,58	5,34	5,71	5,37	5,53	5,58	5,24	5,38
3139	HLA-L	major histocompatibility complex, class I, L (pseudogene)	5,87	6,15	5,97	5,62	5,88	5,93	5,43	5,84	5,88	6,12	6,13	5,64	5,88	5,88
28475	IGHJ6	immunoglobulin heavy joining 6	8,75	8,37	10,97	8,32	7,91	9,95	7,47	7,24	8,55	8,30	10,73	7,76	7,51	9,68
28468	IGHV1-18	immunoglobulin heavy variable 1-18	7,53	8,37	7,97	5,38	5,25	3,83	4,69	3,15	7,29	7,26	6,54	5,11	5,61	4,66
50615	IL21R	interleukin 21 receptor	4,64	4,75	4,85	3,66	3,64	3,71	3,91	4,01	4,05	4,22	4,43	3,71	3,93	4,04
1848	DUSP6	dual specificity phosphatase 6	5,81	5,92	5,71	3,93	3,61	3,90	4,24	3,93	4,57	4,24	4,52	4,22	3,91	4,24

59338	PLEKHA1	pleckstrin homology domain containing A1	6,27	6,04	6,04	4,60	4,34	4,72	5,04	4,65	5,21	4,85	5,22	5,07	4,70	5,07
100131691	MZF1-AS1	MZF1 antisense RNA 1	4,25	4,32	4,29	3,82	3,95	3,93	4,06	4,08	4,16	4,18	4,17	4,01	4,05	4,00
56941	HMCES	5-hydroxymethylcytosine (hmC) binding, ES cell-specific	5,38	5,64	5,84	4,90	4,90	4,84	4,89	5,03	5,17	5,21	5,27	5,01	5,01	5,13
605	BCL7A	BCL tumor suppressor 7A	7,75	7,26	7,77	6,30	5,59	6,30	6,16	5,51	6,77	6,23	6,78	6,48	5,91	6,50
11213	IRAK3	interleukin 1 receptor associated kinase 3	4,21	3,23	4,81	2,44	2,39	2,58	2,63	2,39	3,18	2,67	3,43	2,83	2,32	3,08
8115	TCL1A	T-cell leukemia/lymphoma 1A	8,28	8,50	8,30	4,03	4,13	4,07	3,97	4,25	5,18	5,33	5,75	4,09	4,29	4,40
6565	SLC15A2	solute carrier family 15 member 2	5,24	4,50	4,05	4,35	3,86	3,60	4,16	3,73	4,67	4,16	4,00	4,42	3,81	3,72
54849	DEF8	differentially expressed in FDCP 8 homolog	5,70	5,92	5,95	5,23	5,31	5,31	4,83	5,17	5,27	5,57	5,53	4,98	5,19	5,18
64781	CERK	ceramide kinase	5,88	5,77	5,62	5,21	5,02	4,99	4,93	4,94	5,45	5,42	5,33	5,10	4,97	4,89
28474	IGHV1-2	immunoglobulin heavy variable 1-2	8,91	8,39	7,99	6,90	5,59	4,76	5,99	5,56	8,41	6,52	6,11	7,20	5,48	5,18
151888	BTLA	B and T lymphocyte associated	6,91	7,30	7,06	6,02	5,48	6,11	5,54	5,08	6,37	5,85	6,40	6,07	5,61	6,12
5826	ABCD4	ATP binding cassette subfamily D member 4	5,26	5,38	5,39	5,19	5,14	5,14	5,13	5,20	5,20	5,20	5,19	5,14	5,12	5,12
10391	CORO2B	coronin 2B	4,94	5,44	5,44	4,06	3,60	4,48	3,58	4,04	4,19	4,17	4,46	3,92	3,86	4,19
100379296	RNY4P13	RNA, Ro-associated Y4 pseudogene 13	4,36	4,03	3,44	3,71	3,24	2,85	3,25	2,67	3,45	2,57	2,63	4,03	3,23	3,44
100286946	LOC100286946	ribosomal protein S3A pseudogene	4,26	4,13	4,32	4,14	4,13	4,37	3,99	3,91	4,26	4,20	4,26	4,36	4,34	4,39
100130548	LOC100130548	uncharacterized LOC100130548	4,46	4,43	4,64	4,02	3,99	4,05	4,38	4,30	4,37	4,63	4,67	4,15	4,35	4,35
56937	PMEPA1	prostate transmembrane protein, androgen induced 1	4,84	4,89	5,02	4,62	4,53	4,54	4,72	4,79	4,77	4,89	4,90	4,68	4,80	4,83
2268	FGR	FGR proto-oncogene, Src family tyrosine kinase	5,78	5,58	5,57	5,28	5,14	5,02	5,23	5,28	5,84	5,81	5,45	5,44	5,48	5,13
374928	ZNF773	zinc finger protein 773	4,16	4,24	4,46	3,86	3,92	4,20	4,21	4,12	4,42	4,45	4,56	4,21	4,21	4,39
100271259	RPS3AP33	ribosomal protein S3a pseudogene 33	3,31	3,08	3,94	2,90	3,47	3,84	4,26	4,00	3,70	3,73	4,06	2,66	2,59	2,89
5143	PDE4C	phosphodiesterase 4C	4,67	4,74	4,75	4,58	4,63	4,67	4,67	4,80	4,67	4,78	4,79	4,57	4,67	4,67
249	ALPL	alkaline phosphatase, liver/bone/kidney	3,99	4,05	4,06	3,91	3,98	4,00	4,23	4,46	4,24	4,33	4,22	4,54	4,67	4,56
84957	RELT	RELT tumor necrosis factor receptor	5,79	6,24	5,95	5,85	6,20	6,10	5,98	6,43	6,16	6,67	6,39	6,02	6,51	6,27
11140	CDC37	cell division cycle 37	7,01	7,07	6,97	7,15	7,04	7,18	7,12	7,23	7,36	7,43	7,44	7,25	7,31	7,35
58516	FAM60A	family with sequence similarity 60 member A	6,22	5,74	5,82	6,97	6,54	7,05	6,46	6,19	6,47	6,00	6,43	6,72	6,22	6,64
51699	VPS29	VPS29, retromer complex component	4,14	3,88	3,94	4,78	4,34	4,61	4,09	4,13	4,21	4,01	4,20	4,37	4,16	4,33
645579	EIF4BP6	eukaryotic translation initiation factor 4B pseudogene 6	7,74	7,37	7,80	8,31	7,93	8,49	7,99	7,47	8,12	7,51	8,00	8,36	7,84	8,30
79893	GGNBP2	gametogenetin binding protein 2	7,21	6,86	6,97	7,40	7,21	7,41	7,43	7,16	7,21	6,80	6,93	7,55	7,13	7,21
1808	DPYSL2	dihydropyrimidinase like 2	4,93	4,87	4,99	6,07	5,87	5,63	5,21	5,18	5,40	5,22	5,22	5,17	4,99	5,04
100271548	RPS10P26	ribosomal protein S10 pseudogene 26	8,47	8,02	8,50	9,06	8,70	9,22	8,79	8,04	8,64	8,08	8,60	8,89	8,26	8,78
6894	TARBP1	TAR (HIV-1) RNA binding protein 1	4,50	4,54	3,83	4,96	5,25	4,54	4,80	4,65	4,46	4,48	4,01	4,70	4,69	4,27

11044	PAPD7	poly(A) RNA polymerase D7, non-canonical	5,62	5,66	5,48	5,99	6,23	6,04	6,22	6,18	5,99	5,99	5,90	6,15	6,13	6,05
169981	SPIN3	spindlin family member 3	3,49	3,31	3,42	3,67	3,83	3,67	3,71	3,76	3,62	3,65	3,65	3,66	3,70	3,69
941	CD80	CD80 molecule	3,74	3,16	2,81	6,55	7,14	6,15	5,90	6,20	5,42	5,80	4,70	5,96	6,29	5,12
3927	LASP1	LIM and SH3 protein 1	6,44	6,43	6,51	7,04	6,93	6,89	6,87	6,80	6,84	6,71	6,70	6,91	6,76	6,75
3684	ITGAM	integrin subunit alpha M	4,31	4,33	4,14	6,82	7,02	6,32	6,41	6,48	6,03	5,73	5,20	6,56	6,32	5,75
5464	PPA1	pyrophosphatase (inorganic) 1	4,60	4,08	4,65	5,75	5,06	5,71	5,22	4,90	5,06	4,54	5,19	5,32	4,77	5,39
81552	VOPP1	vesicular, overexpressed in cancer, prosurvival protein 1	5,52	5,68	5,53	6,34	6,67	6,36	6,35	6,59	6,01	6,16	5,94	6,15	6,32	6,09
51808	PHAX	phosphorylated adaptor for RNA export	3,82	3,21	3,62	5,45	4,98	5,58	5,47	4,54	4,69	4,00	4,70	5,18	4,41	5,19
5862	RAB2A	RAB2A, member RAS oncogene family	6,22	5,71	6,00	6,57	6,12	6,58	6,50	5,96	6,30	5,85	6,24	6,47	5,97	6,40
10213	PSMD14	proteasome 26S subunit, non-ATPase 14	4,57	4,38	4,34	5,09	4,99	5,24	5,19	4,82	4,85	4,58	4,84	5,13	4,85	5,08
5552	SRGN	serglycin	5,03	5,34	5,88	6,04	5,86	6,38	5,80	5,83	5,76	6,04	6,50	5,71	6,00	6,46
54915	YTHDF1	YTH N6-methyladenosine RNA binding protein 1	6,21	5,59	6,03	6,46	5,90	6,41	6,54	5,80	6,43	5,98	6,39	6,56	6,13	6,51
169200	TMEM64	transmembrane protein 64	4,70	4,15	4,38	5,35	4,51	4,95	5,18	4,58	5,32	4,55	4,97	5,54	4,78	5,14
102465502	MIR6835	microRNA 6835	3,89	4,20	4,01	4,72	4,88	4,96	4,49	4,92	4,24	4,73	4,84	4,83	5,33	5,32
644330	TPM3P5	tropomyosin 3 pseudogene 5	5,77	5,58	5,93	6,24	6,24	6,23	6,10	5,92	6,27	5,97	6,25	6,49	6,14	6,44
266783	PSMD2P1	proteasome 26S subunit, non-ATPase, 2 pseudogene 1	5,38	5,29	5,32	5,81	5,62	5,61	5,56	5,64	5,68	5,51	5,52	5,76	5,60	5,60
135112	NCOA7	nuclear receptor coactivator 7	3,69	3,47	3,94	4,94	5,05	5,04	4,80	4,62	4,67	4,43	4,49	5,06	4,83	4,87
81611	ANP32E	acidic nuclear phosphoprotein 32 family member E	5,32	5,15	5,24	5,70	5,59	5,91	5,65	5,48	5,69	5,42	5,59	5,86	5,59	5,76
160365	CLECL1	C-type lectin like 1	2,63	2,63	2,77	3,85	3,71	3,65	4,01	3,48	3,85	3,36	3,54	4,32	3,73	3,91
6566	SLC16A1	solute carrier family 16 member 1	4,28	4,33	4,34	5,05	5,24	5,12	5,05	5,15	4,96	4,90	5,09	5,23	5,19	5,36
55299	BRIX1	BRX1, biogenesis of ribosomes	5,89	5,34	5,56	6,37	5,93	6,43	6,33	5,97	6,26	5,65	6,11	6,59	5,99	6,46
84248	FYTTD1	forty-two-three domain containing 1	6,08	5,44	5,94	6,27	6,05	6,84	6,83	6,44	6,60	6,03	6,82	7,10	6,51	7,25
54165	DCUN1D1	defective in cullin neddylation 1 domain containing 1	5,05	4,67	5,07	5,25	5,23	5,43	5,71	5,28	5,20	4,99	5,28	5,60	5,30	5,70
55176	SEC61A2	Sec61 translocon alpha 2 subunit	4,97	5,13	4,51	5,26	5,59	4,96	5,49	5,49	5,24	5,37	4,83	5,43	5,62	5,06
646981	PTPN2P1	protein tyrosine phosphatase, non-receptor type 2 pseudogene 1	4,49	4,92	4,46	5,08	5,35	5,17	5,43	5,43	5,12	5,29	4,84	5,54	5,61	5,28

*LME ANOVA expression−filtered FDR $q < 0.001$ and IgM_{hi} *vs.* IgM_{lo} $p < 0.01$

Supplementary Table 3. Percentages of shared IGH clonotypes per donor. Numbers and percentages of IGH defined clonotypes shared between B cell subsets per donor. Clonally related sequences were identified based on 100% HCDR3 nucleotide identity, and same usage of *IGHV* and *IGHJ* gene usage. Total, unique, and shared clonotypes for each subset of B cells per donor are shown. Percentage is shown in parenthesis.

Supplementary Table 3. Frequencies and percentages of shared IGH clonotypes per donor

Donor 1

Group	Number of Shared clonotypes	Percentage of shared clonotypes			
		Naïve	IgM$_{lo}$	IgM$_{hi}$	IgM Only
IgM$_{lo}$ IgM only IgM$_{hi}$	2		0.31	0.16	0.38
IgM$_{lo}$ IgM$_{hi}$	13		1.99	1.07	
IgM$_{lo}$ IgM only	2		0.31		0.38
IgM Only IgM$_{hi}$	24			1.98	4.62
Total of shared clonotypes (%)	84	0	17 (2.60)	39 (3.21)	28 (5.38)
Total of unique clonotypes (%)	2887	583 (100)	637 (97.40)	1175 (96.79)	492 (94.62)
Total of clonotypes	2971	583	654	1214	520

Donor 2

Group	Number of Shared clonotypes	Percentage of shared clonotypes			
		Naïve	IgM$_{lo}$	IgM$_{hi}$	IgM Only
IgM$_{lo}$ IgM$_{hi}$	4		0.55	0.43	
Total of shared clonotypes (%)	8	0	4 (0.55)	4 (0.43)	0
Total of unique clonotypes (%)	2567	884 (100)	720 (99.45)	921 (99.57)	42 (100)
Total of clonotypes	2575	884	724	925	42

Donor 3

Group	Number of Shared clonotypes	Percentage of shared clonotypes			
		Naïve	IgM$_{lo}$	IgM$_{hi}$	IgM Only
IgM$_{lo}$ IgM$_{hi}$	5		5.15	0.42	
IgM Only IgM$_{hi}$	31			2.58	6.77
IgM$_{hi}$ Naïve	1	0.08		0.08	
Total of shared clonotypes (%)	74	1 (0.08)	5 (5.15)	37 (3.08)	31 (6.77)
Total of unique clonotypes (%)	2993	1308 (99.92)	92 (94.85)	1166 (96.92)	427 (93.23)
Total of clonotypes	3067	1309	97	1203	458
